# DNA-Methylation Signatures of Tobacco Smoking in a High Cardiovascular Risk Population: Modulation by the Mediterranean Diet

**DOI:** 10.3390/ijerph20043635

**Published:** 2023-02-18

**Authors:** Rebeca Fernández-Carrión, José V. Sorlí, Eva M. Asensio, Eva C. Pascual, Olga Portolés, Andrea Alvarez-Sala, Francesc Francès, Judith B. Ramírez-Sabio, Alejandro Pérez-Fidalgo, Laura V. Villamil, Francisco J. Tinahones, Ramon Estruch, Jose M. Ordovas, Oscar Coltell, Dolores Corella

**Affiliations:** 1 Department of Preventive Medicine and Public Health, School of Medicine, University of Valencia, 46010 Valencia, Spain; 2 CIBER Fisiopatología de la Obesidad y Nutrición, Instituto de Salud Carlos III, 28029 Madrid, Spain; 3 Oncology Service, Sagunto Hospital, 46520 Valencia, Spain; 4 Department of Medical Oncology, University Clinic Hospital of Valencia, 46010 Valencia, Spain; 5 Biomedical Research Networking Centre on Cancer (CIBERONC), Health Institute Carlos III, 28029 Madrid, Spain; 6 INCLIVA Biomedical Research Institute, 46010 Valencia, Spain; 7 Department of Physiology, School of Medicine, University Antonio Nariño, Bogotá 111511, Colombia; 8 Department of Endocrinology and Nutrition, Virgen de la Victoria University Hospital, Instituto de Investigación Biomédica de Málaga (IBIMA), University of Málaga, 29590 Málaga, Spain; 9 Department of Internal Medicine, Institut d’Investigacions Biomèdiques August Pi Sunyer (IDIBAPS), Hospital Clinic, University of Barcelona, 08036 Barcelona, Spain; 10 Nutrition and Genomics Laboratory, JM-USDA Human Nutrition Research Center on Aging, Tufts University, Boston, MA 02111, USA; 11 Nutritional Control of the Epigenome Group, Precision Nutrition and Obesity Program, IMDEA Food, UAM + CSIC, 28049 Madrid, Spain; 12 Department of Computer Languages and Systems, Universitat Jaume I, 12071 Castellón, Spain

**Keywords:** DNA methylation, tobacco smoking, EWAS, Mediterranean diet, precision health, epigenetic biomarkers, dietary modulation

## Abstract

Biomarkers based on DNA methylation are relevant in the field of environmental health for precision health. Although tobacco smoking is one of the factors with a strong and consistent impact on DNA methylation, there are very few studies analyzing its methylation signature in southern European populations and none examining its modulation by the Mediterranean diet at the epigenome-wide level. We examined blood methylation smoking signatures on the EPIC 850 K array in this population (n = 414 high cardiovascular risk subjects). Epigenome-wide methylation studies (EWASs) were performed analyzing differential methylation CpG sites by smoking status (never, former, and current smokers) and the modulation by adherence to a Mediterranean diet score was explored. Gene-set enrichment analysis was performed for biological and functional interpretation. The predictive value of the top differentially methylated CpGs was analyzed using receiver operative curves. We characterized the DNA methylation signature of smoking in this Mediterranean population by identifying 46 differentially methylated CpGs at the EWAS level in the whole population. The strongest association was observed at the cg21566642 (*p* = 2.2 × 10^−32^) in the 2q37.1 region. We also detected other CpGs that have been consistently reported in prior research and discovered some novel differentially methylated CpG sites in subgroup analyses. In addition, we found distinct methylation profiles based on the adherence to the Mediterranean diet. Particularly, we obtained a significant interaction between smoking and diet modulating the cg5575921 methylation in the AHRR gene. In conclusion, we have characterized biomarkers of the methylation signature of tobacco smoking in this population, and suggest that the Mediterranean diet can increase methylation of certain hypomethylated sites.

## 1. Introduction

In the past twenty years, data from the Human Genome Project and the advent of omics sciences have revolutionized biomedical sciences, boosting the potential to investigate the molecular mechanisms of diseases [1,2,3]. This effort impacted the rules of research, the methodology of biological discovery, and biomedical research computerization [4,5,6,7]. Therefore, we currently have new genomic tools to investigate novel biomarkers for monitoring health and risk of disease, as well as a great opportunity to translate genomics from the research field to clinical care [8,9]. This has accelerated progress toward so-called “precision medicine” [10,11,12]. However, additional research is still needed to accomplish its predictive and personalized promises. Furthermore, the term “precision health” encompasses wider approaches that occur outside the clinical setting, such as disease prevention, health promotion efforts, and the delivery of individualized health interventions to the appropriate individuals at the proper time [13,14,15]. To achieve precision health, it is necessary to conduct exhaustive research of an individual’s conditions and biomarkers using a variety of measurement technologies. Moreover, it is important to bear in mind that a person’s risk of developing a disease is affected not just by their genome (genetic susceptibility) but also by the exposure to environmental factors (so-called exposome in a wide-range definition) [16,17,18]. In terms of DNA biomarkers, in addition to biomarkers based on DNA sequence changes, such as single nucleotide polymorphisms (SNPs), epigenomic biomarkers are currently of great interest [19,20]. They do not involve changes in the DNA sequence, but rather a series of epigenetic marks of varying types [21,22]. These epigenetic modifications can also have an important effect on gene expression and several of them have been associated with a higher risk of disease [23,24]. The epigenetic mark that has been studied the most is DNA methylation (transfer of a methyl group from S-adenosyl methionine to cytosine residues at the carbon 5 position (5-methylcytosine [5-mC]), mainly occurring in the context of cytosine-phosphate-guanine (CpG) dinucleotides [25]. Moreover, DNA methylation in a number of CpGs is a dynamic epigenetic change [26,27]. Several studies have shown that these DNA methylation marks are important regulators of relevant functions and are linked to the risk of disease [28]. Numerous epidemiological studies have reported associations between various DNA methylation sites and the risk of cardiovascular disease, diabetes, obesity, cancer, and neurodegenerative diseases, among others [29,30,31,32,33,34,35]. In spite of this, the results of the CpG sites found in the various studies continue to be inconclusive; indicating that more research is necessary.

In addition to the risk of disease, differential DNA methylation has been related to environmental exposures [36,37,38]. Currently, it has been estimated that, of all the commonly investigated environmental exposures, tobacco smoking has the most significant impact on DNA methylation [39,40]. Multiple studies carried out in different populations have found that exposure to tobacco smoke is associated with hypomethylation of various CpG sites, with highly statistically significant differences between smokers and non-smokers [41,42,43,44,45,46,47,48,49,50,51,52,53,54,55]. In addition, it has been demonstrated repeatedly that ex-smokers are capable of recovering the demethylation caused by tobacco consumption [41,44,56,57]. Despite differences in methodology, the results of these investigations have been very consistent in identifying dozens of genes with hypomethylated CpG sites in smokers [41,42,43,44,45,46,47,48,49,50,51,52,53,54,55,56,57]. The following are the most prominent of these differentially methylated genes: F2RL3 (F2R like thrombin or trypsin receptor 3), AHRR (aryl-hydrocarbon receptor repressor), PRSS23 (serine protease 23), RARA (retinoic acid receptor alpha), LRRN3 (leucine rich repeat neuronal 3), GFI1 (growth factor independent 1 transcriptional repressor) and GPR15 (G protein-coupled receptor 15). The ranking of other significantly methylated genes or differentially methylated intergenic CpG sites varies between studies based on the characteristics of the population and the coverage of the technology employed to assess DNA methylation [58,59,60]. The majority of the cited research analyzed the methylome using epigenome-wide DNA methylation microarrays [58,61]. In recent decades, the coverage of these DNA microarrays has increased dramatically. The initial Illumina Human array measured methylation at about 27,000 CpGs (27 K). Later, the epigenetics community utilized the HumanMethylation450 (450 K) array for the vast majority of published studies on smoking [58]. More recently, genomic coverage has been increased to over 850,000 CpG sites with the MethylationEPIC (850 K) array [62]. Despite the consistency in detecting hypomethylation in smokers, there have been reports of ethnic differences [63] that would be interesting to investigate further in order to identify more population-specific biomarkers, especially when considering the underrepresented populations of southern Europe [64,65]. Moreover, although it is known that diet might affect DNA methylation [66,67,68,69], none of the large epidemiological studies cited have evaluated the effect of dietary patterns on the differences of DNA methylation between smokers and nonsmokers at the epigenome-wide level [41,42,43,44,45,47,48,49,50,51,52,53,55,56,57]. Our hypothesis is that the methylome signature of tobacco smoking in a southern European population will share characteristics with those of other populations for CpGs with more consistent effects. There will be, however, differences in other methylation sites that can be influenced by a greater adherence to the Mediterranean diet. Therefore, our aims were: (1) to investigate the DNA methylation signatures of tobacco smoking in subjects at high cardiovascular risk from a southern European population including current, former, and never smokers in an epigenome-wide association study (EWAS) using the high-coverage Human Methylation Epic (850 K); (2) to conduct a functional enrichment analysis of the differentially methylated sites associated with tobacco smoking to better understand the pathways and biological processes; and (3) to explore how the Mediterranean diet’s level of adherence modulated the DNA methylation effects of smoking in this population.

## 2. Materials and Methods

### 2.1. Study Design and Participants

We conducted an EWAS on 414 white southern European participants from Spain, aged 55 to 75 years with metabolic syndrome, comprising never, former, and current smokers. These high cardiovascular-risk individuals were recruited for the PREDIMED-Plus-Valencia study, one of the field sites for the ongoing multi-center PREDIMED-Plus trial [70]. These participants were recruited from the primary health care centers of the Valencia region. This region is situated on the eastern coast of the Mediterranean in Spain. At baseline, participants were community-dwelling people (men, 55–75 years; women, 60–75 years) with a body mass index (BMI) between 27 and 40 kg/m^2^ and at least three components of metabolic syndrome [70]. Although the total number of participants recruited at our field center was 465, only 414 subjects were analyzed in this study because this was the final number of individuals who agreed to participate in the genomics/epigenomics studies and whose DNA samples and methylation workflows passed EWAS quality controls. For the remaining variables, there were no significant differences between these subjects and the whole sample at baseline. Participants provided written informed consent, and study protocols and procedures were approved in compliance with the Helsinki Declaration by the Valencia University’s Human Research Ethics Committee (ethical approval code H1373255532771, 15 July 2013; and H1509263926814, 6 November 2017).

### 2.2. Baseline Anthropometric, Clinical and Biochemical Variables

At baseline, anthropometric data and blood pressure were measured by qualified personnel using the PREDIMED-Plus operating protocol [70]. Using calibrated scales and a wall-mounted stadiometer, height and weight were measured. The BMI was computed by dividing weight in kilograms by height in meters squared. Obesity was defined as having a BMI ≥ 30 kg/m^2^. Blood pressure was measured using a validated semiautomatic oscillometer (Omron HEM-705CP, Netherlands). After a 12-h overnight fast, blood samples were collected and fasting plasma glucose, total cholesterol, HDL-C, LDL-C, and triglyceride concentrations were determined as previously reported [71]. We also determined complete blood count (CBC) in venous blood samples. The CBC included total leukocyte counting as well as the types of white blood cells (neutrophils, eosinophils basophils, monocytes, and lymphocytes). For these determinations, anticoagulated blood samples were processed fresh and measured within a few hours after collection in our reference clinical laboratory at the University Clinic Hospital (Valencia) using automated hematology analyzers [72]. Medication use was assessed at baseline as reported [70]. Type 2 diabetes was defined as previous clinical diagnosis of diabetes, HbA1c levels ≥ 6.5% or use of anti-diabetic medication [70].

### 2.3. Tobacco Smoking and Adherence to the Mediterranean Diet

Self-reported smoking status was obtained by a previously described questionnaire administered by trained staff [70]. This questionnaire contained a general question regarding current and past tobacco usage (never smoker, former smoker of more than five years, former smokers of five to one years, former smokers of less than one year, and current smokers). In addition, questions regarding the type of tobacco smoked (cigarettes, cigars, and pipe), as well as the number of cigarettes/cigars/pipes smoked per day, and the average number of years the participant had smoked were included. Current smokers were classified as those who smoked at least one cigarette, cigar, or pipe per day. None of the participants indicated that they smoked pipes. However, six participants reported smoking cigars. Taking into account that the nicotine number of cigars is higher than that found in a cigarette, a conversion factor was employed to consider this weight [73]. Therefore, on average, we considered that one cigar was equivalent to three cigarettes, and all smokers were analyzed together. For current smokers, we also analyzed the average number of cigarettes smoked per day and calculated the number of pack-years smoked as the number of cigarettes smoked per day divided per 20 and multiplied by the number of years the participants have smoked [74]. Smoking status was first analyzed as an ordinal variable including five levels (never, former > 5 years, former 5–1 years, former < 1 year and current smokers). The second smoking status was defined in three categories: current smokers, ex-smokers, and non-smokers. Finally, never smokers were compared to current smokers.

Total leisure-time physical activity-related energy expenditure was estimated as the sum of frequency, duration, and intensity of each activity divided by 30 days/month (METmin/day) using the validated REGICOR questionnaire [75].

Adherence to the Mediterranean diet was assessed by the validated PREDIMED-Plus 17-item score [76], and updated version of the previously validated PREDIMED 14-item scale [77]. The 17-item questionnaire included 17 questions related to Mediterranean diet. The questionnaire was scored with 1 point for each item capturing adherence to the Mediterranean diet and 0 points for items that did not: use only extra virgin olive oil for cooking, salad dressings, and spreads; fruits; vegetables; white bread; whole bread; red meat or meat products; butter, margarine or cream; sugary beverages; legumes; fish or shellfish; commercial sweets or pastries; nuts; chicken, turkey, or rabbit; sofrito (sauce made with tomato and onion, garlic and olive oil); add preferentially non-caloric artificial sweeteners to beverages instead of sugar; non-whole grain pasta or white rice; moderate red wine consumption. A higher score (from 0 to 17) indicated greater adherence to the Mediterranean diet. As previously reported [78], the score was then categorized into two groups reflecting low (from 0 to 8 points) and high adherence (from 9 to 17 points) to the Mediterranean diet.

### 2.4. DNA Isolation and DNA-Methylation Analysis

Genomic DNA was isolated from blood at baseline as previously reported [78]. The quantity of double-stranded DNA was measured using PicoGreen (Invitrogen Corporation, Carlsbad, CA, USA). Only samples providing 500 ng of high-quality DNA were processed further for epigenome-wide methylation analysis. For methylation profiling, we used the Infinium HumanMethylationEPIC BeadChip (850 K) array (Illumina, San Diego, CA, USA), which interrogates over 850,000 CpG sites for DNA methylation profiling, including more than 90% of the probes on the 450 K array and additional CpG-sites [79]. The positions (sample wells) of the DNA samples were randomized on the microchips to minimize batch effects [80,81]. We created a random list of sample numbers and placed the samples on the chip based on the random list. Further processing of the arrays was performed at the Human Genomics Facility, Erasmus MC, Rotterdam.

Bisulfite conversion of DNA was performed using the Zymo EZ-96 DNA Methylation Kit (Zymo Research, Irvine, CA, USA) and samples were hybridized to the Illumina EPIC array, according to the manufacturer’s protocol. Microarrays were scanned with an Illumina HiScan system and “.idat” files were generated. Quality control procedures were implemented at Human Genomics facility to assess the quality and reliability of the generated DNA methylation data, involving the use of Minfi, Meffil and ewastools R packages [82,83,84]. Briefly, this quality control identified samples that failed or had sub-optimal control metrics regarding poor bisulfite conversion, all types of poor hybridization, and samples with a low call rate. A total of 414 samples passed this quality control and were further analyzed for the EWAS. Moreover, in this pre-processing quality control, a subset of probes in these samples were identified as suboptimal, and we created a list for further filtering.

Additional DNA quality checks, data normalization, and filtering were performed with the Partek^®^ Genomics Suite^®^ [85]. Probes from the X and Y chromosomes (due to the analytical complexities associated with sex chromosome dosage differences between XX and XY individuals), as well as low-quality probes were filtered and excluded. Functional normalization, a method that used the internal control probes present on the array to infer between array-technical variation and that extends quantile normalization and outperforms other types of normalization previously used [86] was carried out. Also, dye correction and normal-exponential out-of-band (NOOB) background correction were applied [87]. Beta-values (ranging from 0 to 1 and) were obtained as metrics to measure methylation levels and are based on the measured intensities of the pair of probes (a methylated probe and an unmethylated probe) at each CpG site [88]. Subsequently, beta-values were converted to M-values as follows: M-value = log_2_ (beta/(1 − beta)). The advantage of the M-values is the higher homoscedasticity compared with beta-values [89]. Therefore, we followed the methodological recommendations and employed M-values for statistical analysis and beta-values for direct biological interpretation (corresponding to the percentage of a CpG site that is methylated) [88,89].

### 2.5. Statistical Analysis and EWAS

To summarize the characteristics of the analyzed sample, descriptive statistical tests were performed. Chi-square tests were used to compare proportions. Student *t*-tests and ANOVA tests were applied to compare crude means of continuous variables. Triglyceride concentrations were log-transformed for statistical testing. To estimate the association between smoking and DNA methylation, we performed several analyses adapted to our specific objectives. As an ordinal variable, we first conducted an EWAS for the smoking phenotype with five levels (never, former > 5 years, former 5–1 years, former 1 year, and current smokers). For statistical testing, genome-wide M-values for methylation at CpG sites were employed. ANCOVA models with covariate adjustments were used to detect differential methylation. Several statistical models were fitted to account for potential confounding variables and ensure consistency. The models were gradually adjusted to account for sex, age, batch effect, BMI, type 2 diabetes, and leukocyte cell counts. Instead of using estimated leukocyte cell counts [90], we used directly measured cell counts (neutrophils, eosinophils basophils, monocytes, and lymphocytes after checking multicollinearity), increasing validity [91,92]. After verifying each step, we only presented the results corresponding to the model adjusted for all covariates. *p*-values and partial regression coefficients for each CpG site were computed. The *p*-value cut-off for EWAS statistical significance was set at *p* < 9 × 10^−8^, as proposed by Mansell et al. [93] when using the EPIC methylation array. *p* < 1 × 10^−5^ was considered as the suggestive level of significance. Manhattan plots of the adjusted EWAS model were computed in R and depicted. For quality control, quantile-quantile (Q-Q) plots comparing the expected and observed *p*-values were performed in the R-statistical environment. Likewise, the genomic inflation factor (lambda) values were computed [94]. For top-ranked differentially methylated CpG sites, beta-values were obtained and plotted against the five levels for the smoker phenotype. Second, to confirm the association between smoking and methylation using the 3-categories variable (never smokers, former smoker groups and current smokers) and to compare never versus current smokers and former smokers versus current smokers, another EWAS was fitted. M-values were used, and multivariate ANOVA models were fitted. Models were adjusted for sex, age, the batch effect, BMI, type 2 diabetes, and leukocyte cell counts. For each contrast, the adjusted *p*-values and the beta differences were generated. Likewise, Manhattan plots for the categorical analyses were depicted.

Third, gene set enrichment analysis of the differentially methylated CpG sites [95,96] was performed for biological and functional interpretation using Partek Genomics Suite and Partek Pathway. Kyoto Encyclopedia of Genes and Genomes (KEGG) pathway analyses and Gene ontology (GO) enrichment [97,98] were conducted for the top differentially methylated CpG sites obtained in the EWAS for tobacco smoking (5 levels of smoking), that passed the false discovery rate (FDR) cut-off. Enrichment scores were computed, and we presented the raw and Bonferroni corrected for multiple comparisons to identify which KEGG pathways and GO terms were significantly enriched.

Fourth, we focused on current smokers and analyzed the associations of the top-ranked CpG sites identified in the association between tobacco smoking (5 levels) and genome-wide methylation with the number of cigarettes smoked per day. Fifth, we tested in this population the best predictors of smoking status (never smokers versus current smokers). We selected the five top-ranked CpG sites in the model analyzing smoking status (5 levels), estimated the sensitivity and specificity of each CpG and explored the predictive value using receiver operative curve (ROC). The area under the curve (AUC), its 95% confidence interval (CI) and *p*-values were computed for each CpG to check the performance of classification models, using SPSS Statistics for Windows Ver. 26 (IBM Corp., Armonk, NY, USA).

Finally, we conducted stratified EWAS by sex on the whole population. The association between tobacco smoking (5-levels) and genome-wide DNA methylation was examined in men and women separately, and adjusted *p*-values and partial correlation coefficients were computed for each stratum. Moreover, we conducted a stratified EWAS depending on the level of Mediterranean diet adherence (based on the mean as previously detailed) to examine the association between tobacco smoking and genome-wide methylation in subjects with a low level of adherence to the Mediterranean diet in comparison with subjects with a high level of adherence. Adjusted *p*-values and correlation coefficients were computed for each CpG site. Furthermore, we depicted the so-called Miami plot [99], which allows for the comparison of two Manhattan plots for both Mediterranean diet adherence strata. Later, we selected the most relevant CpG sites and analyzed the interaction term between smoking (3 categories) and adherence to the Mediterranean diet (2 levels) on the CpG site methylation levels (beta-values) in a hierarchical multivariate model. For analyses involving selected CpGs, a *p*-value < 0.05 (two-sided) was considered statistically significant.

## 3. Results

### 3.1. Participants Characteristics

Table 1 shows the demographic, anthropometric, clinical, biochemical and lifestyle characteristics of the 414 participants analyzed for EWAS. All subjects participated in the PREDIMED Plus-Valencia study, and baseline measurements were performed on all parameters. They were elderly men and women with metabolic syndrome (mean age 65 ± 0.2 years). In the whole population, the prevalence of never, former, and current smokers was 45.4%, 43.0%, and 11.6%, respectively. In addition, we considered three types of former smokers based on the length of time since cessation (more than 5 years, between 5 and 1 year and less than 1 year). There were statistically significant differences by sex (*p* < 0.001), with men having a greater proportion of current smokers (16.1%) than women (7.9%); *p* < 0.001. The quantity of cigarettes smoked per day and the duration of smoking did not differ significantly by sex (*p* < 0.05) in current smokers.

### 3.2. Association between Tobacco Smoking (5 Levels) and Its Epigenome-Wide Methylation Signatures

**Figure 1:**
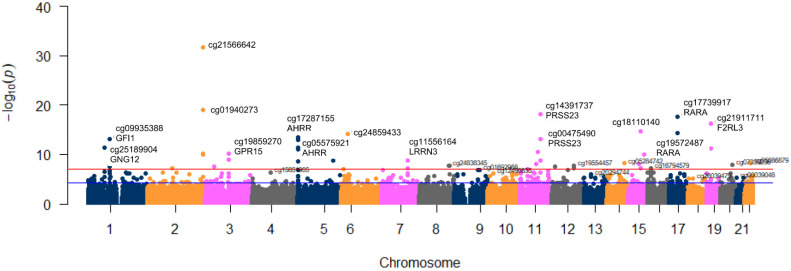
Manhattan plot of epigenome-wide associations (EWAS) for tobacco smoking (5 levels) adjusted for covariates in the whole sample. Red line represents the EWAS significance level *p* = 9 × 10^−8^. The blue line represents *p* = 1 × 10^−5^. The GRCh37/hg19 assembly has been used.

**Figure 2:**
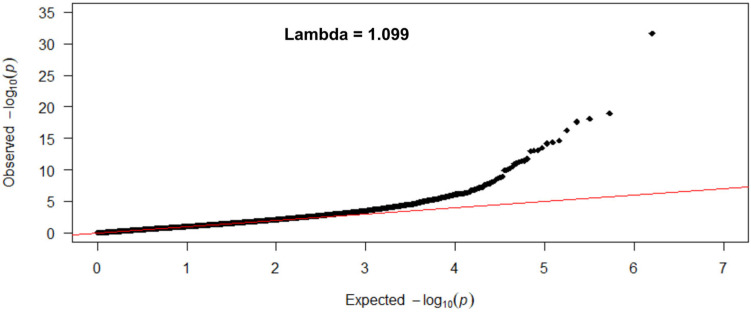
Quantile-quantile (QQ) plot for CpG-site association in the EWAS for smoking (5 levels) in Figure 1.

We first considered five levels of tobacco smoking (never, former > 5 years, former 5–1 years, former 1 year, and current smokers) as a quantitative variable and analyzed the DNA methylation signatures of tobacco smoking in an EWAS using the EPIC 850 K methylation array. Models were adjusted for sex, age, the batch effect, BMI, type 2 diabetes, and leukocyte cell counts. Figure 1 shows the Manhattan plot corresponding to the adjusted *p*-values for each analyzed CpG site (M-values) in the EWAS. Several differentially methylated CpG sites surpassed the epigenome-wide significance threshold *p* < 9 × 10^−8^ [93]. Figure 2 depicts the QQ plot corresponding to this EWAS.

No signal inflation was seen in this study (lambda = 1.099). Table 2 provides information on the differentially methylated CpG sites, genes, corrected *p*-values, and partial regression coefficients obtained for the statistically significant top-ranked positions (n = 46) in this EWAS (We have included a glossary with the annotated gene symbols and names in Table S1).

We observed a linear trend, from never smokers to former and current smokers, of decreased methylation across the five smoking levels defined for these 46 CpG sites. The additional information indicating whether or not the CpG site was present in the Illumina 450 K array shows that only 13 CpGs were profiled by the EPIC 850 K array. The strongest association was observed at cg21566642 (*p* = 2.2 × 10^−32^). This CpG site (represented on both the 450 K array and the EPIC 850 K array) is intergenic and located in the 2q37.1 region close to the alkaline phosphatase, placental-like 2 (ALPPL2) gene.

The genetic environment where the cg21566642 is located is very relevant (Figure S1). According to the University of California Santa Cruz (UCSC) browser and using the GRCh38/hg38 assembly, this site is located in a CpG island (CGI) but also in a promoter-like signature region according to ENCODE (Encyclopedia of DNA Elements).

**Figure 3:**
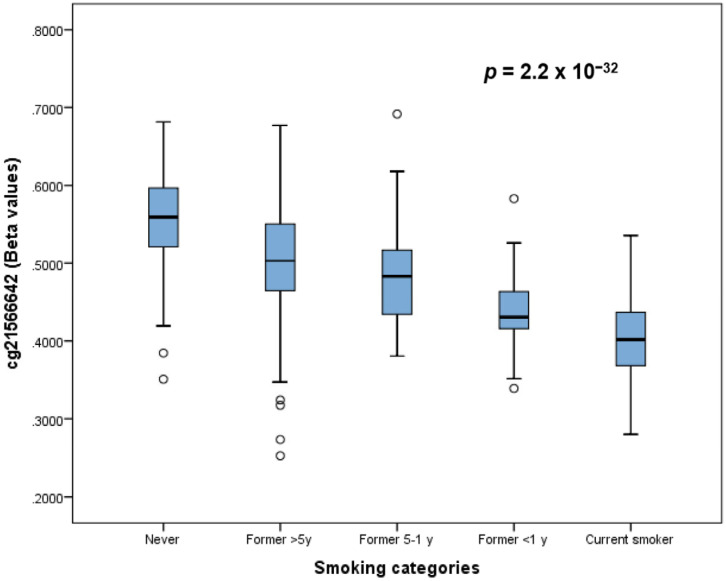
Box plots of distributions showing methylation (beta-values) of the cg21566642 site (chromosome 2) by smoking status (5 levels). The box plots show the five-number summary of a set of data: the minimum score, first (lower) quartile, median, third (upper) quartile, and maximum score.

Figure 3 shows the box plots of the corresponding methylation beta-values for the cg21566642 site according to the smoking status (5 levels) revealing the dose–response association between the smoking phenotype and DNA hypomethylation.

This result is highly consistent and has been observed previously in several studies [43,48,55,61]. Likewise, the second hit was cg1940273 (*p* = 1.2 × 10^−19^), also located in the 2q37.1 region, and reported in previous studies [43,48,55,61]. cg14391737 was the third-most outstanding CpG site in terms of significance. This CpG site was only profiled by the EPIC array, hence prior studies that utilized the 450 K array were unable to discover it. Nevertheless, the PRSS23 gene, in which cg14391737 is annotated, is one of the genes most consistently associated with differential DNA methylation in tobacco smokers, as revealed by different kinds of arrays. Similarly, all the other genes listed in Table 2 (except the NUDT4P2: nudix hydrolase homolog 4 pseudogene 2) have been consistently reported in other studies conducted in other populations, thus the methylation signature of tobacco smoking in this Spanish Mediterranean population was, in general, quite similar. In addition to PRSS23, we detected: RARA, F2RL3, AHRR, GFI1,GNG12 (G protein subunit gamma 12), ARRB1 (arrestin, beta 1), GPR15, ANPEP (alanyl aminopeptidase), C5orf62 (small integral membrane protein 3), LRRN3 (leucine rich repeat neuronal 3), MGAT3 (beta-1,4-mannosyl-glycoprotein 4-beta-N-acetylglucosaminyltransferase), ITPK1 (Inositol 1,3,4-trisphosphate 5/6-kinase family protein), LRP5 (LDL receptor related protein 5), ATP9A (ATPase phospholipid transporting 9A putative), MTSS1 (MTSS I-BAR domain containing 1), NUDT4 (nudix hydrolase homolog 4), ETV6 (ETS variant transcription factor 6), UXS1 (UDP-glucuronic acid decarboxylase 1), XYLT1 (xylosyltransferase 1); SEMA7A (semaphorin 7A-John Milton Hagen blood group) and LMO2 (LIM domain only 2). Also, the intergenic CpG sites have been linked to differential methylation in several previous studies. Nonetheless, the order of statistical significance of each CpG differs the most from study to study. In this Mediterranean population, for example, cg05575921 in the AHRR gene did not rank as significantly as in other studies.

### 3.3. Association between Tobacco Smoking (Comparing Categories) and Its Epigenome-Wide Methylation Signatures

**Figure 4:**
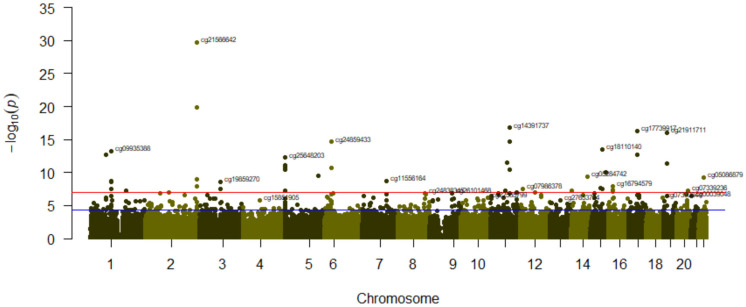
Manhattan plot of the epigenome-wide association among never smokers relative to current smokers (multivariate adjusted). Red line represents the EWAS significance level *p* = 9 × 10^−8^. The blue line represents *p* = 1 × 10^−5^. The GRCh37/hg19 assembly has been used.

Further, in a sensitivity analysis, we examined the consistency of the previous results (which considered 5 levels of tobacco smoking and were analyzed as a linear variable) by examining tobacco smoking as a categorical variable with three categories (never, former, and current smokers). In addition, we examined the DNA methylation signature of never smokers versus current smokers (Figure 4) using an EWAS that was adjusted for sex, age, batch effect, BMI, type 2 diabetes, and leukocyte cell counts.

Part A of Table 3 ranks the 15 most differentially methylated CpG sites according to their *p*-value for the categorical variable tobacco smoking (3 categories). Table S3 presents the complete list of statistically significant CpG sites for this association. The results were highly consistent with those provided for smoking as a five-level quantitative variable. Likewise, similar but most significant results were obtained when never smokers were compared with current smokers (Table 3, part B). We detected 43 CpGs that were differently methylated at EWAS level between never-smokers and smokers. Intergenic cg21566642 (*p* = 1.68 × 10^−30^) in the 2q37.1 region was confirmed as the hit. Focusing on beta differences, never smokers had an average of 14.3% greater methylation levels at this position than never smokers. All statistically significant methylation sites for this comparison have been previously reported in other studies, which confirm the consistency of our findings with those of previous research.

**Figure 5:**
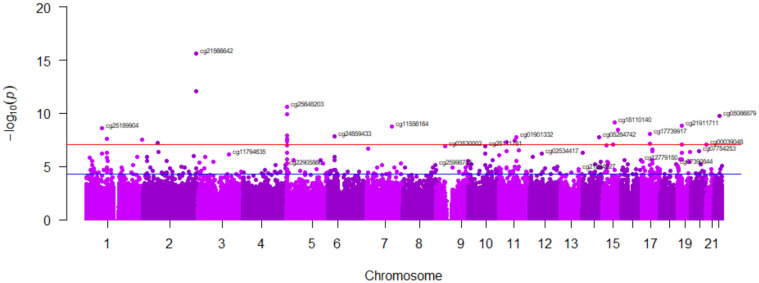
Manhattan plot of the epigenome-wide association among former smokers relative to current smokers (multivariate adjusted). Red line represents the EWAS significance level *p* = 9 × 10^−8^. The blue line represents *p* = 1 × 10^−5^. The GRCh37/hg19 assembly has been used.

In addition, we examined DNA methylation signatures of former and current smokers at the EWAS level (Figure 5).

The 15 most differentially methylated CpGs for this comparison are given in Table 4, along with their respective beta differences (Table S4 presents the complete list). We identified 24 differently methylated sites between former and current smokers at the EWAS level.

Former smokers exhibited greater methylation values than current smokers across every position. Results were, in general, similar to previous findings, but we discovered a novel statistically significant (*p* = 2.8 × 10^−8^) differentially methylated CpGs for the comparison between former and current smokers reported for the first time here. It was the cg2093781 (only profiled in the EPIC 850 K), located in chromosome 1 and annotated to the KIP26B (kinesin family member 26 B) gene. Interestingly, we discovered another novel differentially methylated GpG site that was borderline significant in the comparison between former and current smokers. It was, cg25094529 (only profiled in the EPIC 850 K), annotated to the SRP14-AS1 (SRP14 antisense RNA1) gene on chromosome 15.

### 3.4. Gene Set Enrichment Analysis of the Differentially Methylated CpG Sites Using KEGG and GO

KEGG pathway analyses and GO functional enrichment were conducted for the top differentially methylated CpG sites obtained in the EWAS for the tobacco smoking (5 levels) that passed the FDR cut-off. Table 5 shows the top-ranked pathway names, the enrichment *p*-values and the Bonferroni corrected enrichment *p*-values. We detected 31 KEGG pathways that passed the Bonferroni correction. Among these most-enriched pathways, 11 related to cancer, including: non-small cell carcinoma, glioma, hepatocellular carcinoma, transcriptional regulation in cancer, small cell lung cancer, pathways in cancer, melanoma, pancreatic cancer, chronic myeloid leukemia, Karposi sarcoma, and bladder cancer. We also detected enrichment in pathways related to osteoporosis, insulin signaling, cardiovascular health, addiction, and circadian rhythm. Table S5 displays the first 22 GO enrichment functions, including their type (biological process, cellular component, or molecular function), enrichment score, raw enrichment *p*-value, and Bonferroni-corrected enrichment *p*-value. Most of the statistically significant findings concerned biological processes, as the hits “Negative modulation of tau-protein kinase activity” (*p* = 1.1 × 10^−50^), followed by “Positive regulation of interleukin-13 production” (*p* = 3.1 × 10^−42^).

### 3.5. Dose-Response of DNA Methylation in Current Smokers

**Figure 6:**
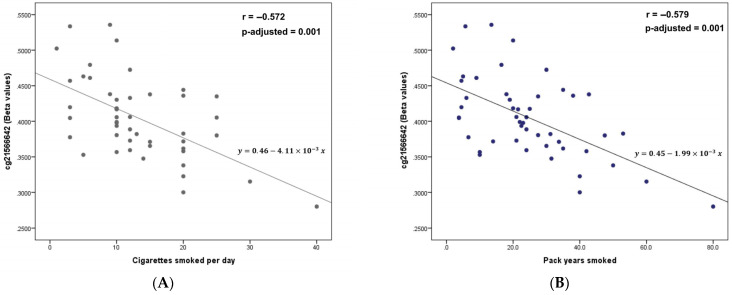
Scatter plot for the association between methylation levels in the cg1566642 site (beta-values) and cigarettes smoked per day (panel **A**), and pack-years smoked (panel **B**) in current smokers (n = 48).

We examined dose–response associations in current smokers for significant CpGs from the EWAS of tobacco smoking (5 levels of smoking status) by correlating methylation (beta-values) in the top-ranked CpG sites and the number of cigarettes smoked. We used both daily cigarettes smoked, and number of pack-years smoked. The scatter plots for the hit cg1566642 site in the 2q37.1 region are depicted in Figure 6. Both the number of cigarettes smoked per day (panel A) and pack-years smoked (panel B) exhibited a robust dose–response relationship. Higher cigarette consumption was substantially linked with decreased methylation at this location. Table S6 shows the correlations of methylation (beta-values) with cigarettes smoked per day and number of pack-years smoked for the other CpGs in Table 2. For the most relevant CpG sites, statistically significant dose–responses in terms of hypomethylation were identified.

### 3.6. Methylation at Selected CpG as Predictors of Smoking Status

We assessed the performance of the five top-ranked CpG sites in Table 2 as classifiers of smoking status. The DNA methylation level (beta-values) at these sites were very good classifiers between current smokers and never smoker. Figure S2 shows the ROC curves for the five top-ranked CpGs and the corresponding AUCs, 95% CI and *p*-values. The cg1566642 site at 2q37.1 was the strongest classifier between smokers and never smokers with an AUC of 0.97; 95%CI: 0.94–0.99; *p* = 1.4 × 10^−23^.

### 3.7. Association between Tobacco Smoking (5 Levels) and Its Epigenome-Wide Methylation Signatures by Sex

We examined sex-specific DNA methylation signatures for tobacco smoking in two separate EWAS carried out in men and women. Analyzed as a five-level variable, tobacco smoking was adjusted for age, batch effect, BMI, type 2 diabetes, and leukocyte counts.

Table 6 displays the top differentially methylated CpGs for each EWAS in both men and women. In general, men and women had similar results on the most relevant CpGs. Thus, cg1566642 in the 2q37.1 region was the hit in both sexes (*p* = 2.2 × 10^−15^; r = −0.561 in men and *p* = 4.1 × 10^−16^; r = −0.522 in women).

Similarly, hypomethylation was identified in the most relevant GpGs for PRSS23, F2RL3, RARA, and AHRR in both men and women. However, additional sex-specific differences can be observed at certain sites. In this population, we discovered a novel CpG site associated (*p* = 9.1 × 10^−9^) with cigarette smoking in women. It corresponds to the SPATA17 (Spermatogenesis Associated 17) gene and is only profiled by the EPIC 850 K array. This association is being reported for the first time at this stage.

Moreover, despite the fact that chromosomes X and Y were excluded from all other analyses, we investigated the effect of tobacco smoking (5 levels) on CpG sites located on chromosomes X and Y in the sex-specific results. Table S7 shows the most significant CpG sites on chromosome X associated with tobacco smoking in women. Likewise, Table S8 shows the most significant CpG sites on chromosomes X and Y associated with tobacco smoking in men. Several differences between the sexes were identified; however, the comparison is difficult due to the XX women and the methodological limitations of X-chromosome inactivation. In terms of differences, we would like to mention that the ACE2 (Angiotensin-converting enzymes 2) gene was in the list of the top-ranked differentially methylated genes associated with tobacco smoking in women. This gene was not significant in men. However, we detected several CpG sites significantly associated with tobacco smoking in both men and women, WWC3 (WWC Family Member 3) being the most consistent.

### 3.8. Modulation of Tobacco Smoking’s Epigenome-Wide Methylation Signature by Adherence to the Mediterranean Diet

**Figure 7:**
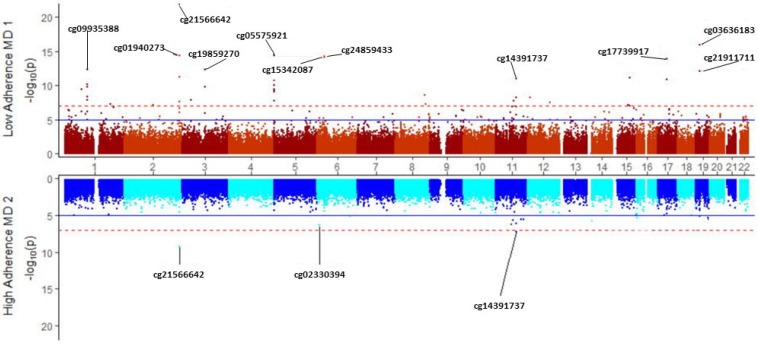
Miami plot for the epigenome-wide methylation analysis for smoking (5 levels), depending on the adherence to the Mediterranean diet: Low adherence (above x-axis) and High adherence (below x-axis). The top 11 most significant CpGs for the low-adherence level are highlighted. For the High adherence level, the 3 most significant CpGs are highlighted. Red line represents the EWAS significance level *p* = 9 × 10^−8^. The blue line represents *p* = 1 × 10^−5^. The GRCh37/hg19 assembly has been used.

We conducted a stratified EWAS for tobacco smoking (5 levels) depending on the level of Mediterranean diet adherence (low and high) to explore the potential modulation of the Mediterranean diet on the tobacco smoking’s epigenome-wide methylation signature. Figure 7 displays the corresponding Miami plot for both EWAS adjusted for sex, age, batch effect, BMI, type 2 diabetes, and leukocyte counts. We detected an apparent different profile. Only cg1566642 in the 2q37.1 and cg4391737 in the PRSS23 gene presented statistically significant EWAS differences in methylation at both the low and high levels of adherence.

Table 7 shows the more information regarding the top differentially methylated CpGs depending on the adherence to the Mediterranean diet, the annotated genes, the *p*-values, and the correlation coefficients.

When adherence to the Mediterranean diet was low, we observed a methylation profile typical of tobacco smoking, with the top-ranked genes consistently reported in other populations. Despite the limited sample size, 38 differentially methylated CpGs were identified at the EWAS level in subjects with a low adherence to the Mediterranean diet. However, fewer significant differences in methylation were detected in individuals with high levels of adherence. This stratum’s relatively small sample size may have an influence on the findings. Nonetheless, the extent of the observed differences in statistical significance and in a subset of the ranked genes revealed a possible effect of the Mediterranean diet on the methylation level of certain CpGs. One of these CpGs additional modulated by the Mediterranean diet could be cg5575921 in the AHRR gene. This site is one among the CpGs most strongly linked to tobacco smoking in previous research. However, when examining its influence on this Mediterranean population as a whole, this CpG did not occupy the top differential methylation levels. After the analysis stratified by adherence to the Mediterranean diet, this CpG was among the top four hits in the low-adherence group but lacked statistical significance in the high-adherence group. In order to assess whether there is a statistical interaction between adherence to the Mediterranean diet and tobacco use in the methylation of specific CpGs, we selected the two most relevant CpGs (cg1566642 in the 2q37.1 region and cg5575931 in the AHRR gene) and examined the statistical significance of the interaction term diet*smoking in a hierarchical model. These findings are shown in Figure S3. Panel A shows that there was no smoking*Mediterranean diet interaction for the cg1566642 site (*p* = 0.808). However, there was a statistically significant interaction (Panel B) for the cg5575921 in the AHRR gene (*p* = 0.013). Figure S3 also shows the statistically significant interaction between smoking and Mediterranean diet adherence on DNA methylation for another two selected CpGs (panels C and D). This is an exploratory study, and additional research is needed to better characterize how the Mediterranean diet modulates methylation by smoking status.

## 4. Discussion

We examined tobacco smoking DNA methylation signatures in blood (including never, former, and current smokers) in an older Spanish-Mediterranean population with metabolic syndrome. This is the first study in this population using the high density EPIC 850 K methylation array. In comparison to other European populations, this population has received relatively little attention in the analysis of epigenomics markers. Therefore, it is informative to obtain data on the characteristics of this population to better understand the external validity of biomarkers reported in other countries [64,65,100]. A previous study conducted in a Mediterranean cohort recruited in Girona profiled DNA methylation associated with tobacco smoking using the 450 K array and differed from the present study in a number of aspects [48]. In the present study, the sample was enriched with former smokers, allowing us to analyze three distinct levels of former smokers based on the length of time since smoking cessation. Similarly, the average number of years that smokers had smoked was very high (38 years). The main EWAS in the whole population considered five levels of smoker status and revealed quantitative effects on DNA methylation of the top-ranked differentially methylated CpG sites. In this EWAS, we detected 46 differentially methylated CpGs at the epigenome-wide level of significance [93]. The strongest association (hypomethylated in smokers) was observed at the intergenic cg21566642 (represented on both the 450 K and the EPIC 850 K methylation arrays) located in the 2q37.1 region, close to the ALPPL2 gene. The genomic “environment” where this CpG site is located in locus with functional relevance (in a CGI but also in a promoter-like signature region). The second hit was cg1940273, also located in the same region. Likewise, the cg21566642 methylation site was previously reported as the top hit by Christiansen et al. [61] analyzing subjects from the United Kingdom using the EPIC 850 K array. Other studies also reported this CpG site as one of the most significant hits [43,48,55,101]. In addition to the ALPPL2 gene, other two genes coding for closely related alkaline phosphatase genes, placental (ALPP) and intestinal (ALPI) are located within the 2q37.1 region. Alkaline phosphatases are known as plasma membrane-bound glycoproteins that have been involved in several diseases including cancer, cardiovascular and inflammatory diseases, among others [102,103]. The mechanisms by which tobacco smoking can lead to hypomethylation of the cg21566642 and other CpGs in this region are not yet well understood. A complex relationship between smoking, immunoglobulin G glycosylation, and DNA methylation at the cg21566642 site has been postulated [104]. However, additional research on these pathways and how hypomethylation at this site is associated with disease risk is required. In addition to the statistically significant hit (cg21566642) in our primary EWAS, we detected as statistically significant another additional 45 differentially methylated CpGs profiled with either the 450 K or EPIC methylation arrays. We used the recently updated “The EWAS Catalog: a database of epigenome-wide association studies” to verify the novelty of these CpGs [105]. All the CpGs discovered in our EWAS as statistically significant in the whole sample had previously been reported in other studies [40,41,42,43,44,45,46,47,48,49,50,51,52,53,54,55,56,57,61,101,105,106,107], except for the cg15533935 in the NUDT4P2 gene (only present in the EPIC 850 K array), which is described for the first time here. Even though the CpGs only found in the EPIC 850 K array are more recent, they have also been reported in previous studies that have used this array [51,55,61,107]. This result confirms the high consistency of the effect of tobacco smoking on DNA methylation in diverse populations and the relevance of genes related to inflammation and immunity pathways (AHRR, F2RL3 and PRSS23, among others) [108,109]. However, it is worth noting that, despite the consistency in detecting various CpGs as statistically significant, the contribution of each of them may differ and be more specific to each population. Consequently, despite obtaining statistically significant results for the previously reported PRSS23, RARA, F2RL3, AHRR, GFI1, GNG12, ARRB1, GPR15, ANPEP, LRRN3, MGAT3, ITPK1, LRP5, ATP9A, MTSS1, NUDT4, ETV6, UXS1, XYLT1, SEMA7A, and LMO2 [105], the rank in this Mediterranean population was distinct and more related to population characteristics (sex, age, smoking status and diverse environmental factors, among others). In the majority of previous studies [105], the cg05575921 site in the AHRR gene has been found to be the first site of differential methylation associated with tobacco smoking. Nevertheless, it rated eleventh in the EWAS for our whole population. This can be explained by taking into account what we discovered when we examined the modulation of methylation in smoking-CpG sites due to Mediterranean diet adherence. The cg05575921 site in the AHRR gene ranked high when adherence to the Mediterranean diet was low in the stratified EWAS considering two adherence strata (low and high). In a hierarchical model adjusted for covariates, we found a statistically significant interaction term between tobacco-smoking status and adherence to the Mediterranean diet in determining DNA-methylation at the cg05575921 site.

A higher adherence to the Mediterranean diet increased the level of methylation in the cg05575921 site in current smokers, helping to counteract the hypomethylation induced by smoking. It is known that diet can have an effect on DNA methylation [66,67,68,69], although this is not well understood. Although this is the first study to focus on the effect of Mediterranean diet on DNA methylation at the genome-wide level, other studies have examined the impact of specific foods/nutrients on the methylation at the CpGs in the AHRR gene depending on the smoking status [110,111]. Thus, Tsuboi et al. [110] reported in a Japanese population that dietary intake of vegetables and fruits rich in provitamin A increased the percentage of AHRR DNA methylation in current smokers. Similarly, Shorey-Kendrick et al. [111], in a clinical trial, reported that supplements of vitamin C during pregnancy prevented offspring DNA hypomethylation in the AHRR gene and other genes associated with maternal smoking. Although our study in the Mediterranean population was exploratory, and more detailed analysis of the effects of the Mediterranean diet in modulating the methylation profile of tobacco smoking is required, the findings are very interesting and may explain the differences found between populations, as well as the varying risks of disease among smokers based on diet. In our exploratory analysis, we also detected that the modulatory effect of the Mediterranean diet was more specific for certain CpGs, such as the mentioned cg05575921 in the AHRR gene, for the cg9936388 in the GFI1 gene or for the cg01901332 in the ARRB1 gene. No significant interaction between adherence to the Mediterranean diet and tobacco smoking was found for the top hit cg21566642 in the 2q37.1 region. This contributes towards explaining the strong association between smoking and methylation at this site in the present study. Likewise, in this population, the cg21566642 site had a very high discriminative capacity to identify current smokers versus never smokers (ROC-AUC = 0.97; 95% CI: 0.94–0.99). In the subgroup analysis comparing the EWAS effects of never smokers versus current smokers, we also found the cg2156664 site as the top hit for statistical significance. For this comparison, we found a similar list of CpGs as statistically significant as when analyzing the entire population, but with some differences in ranking and sites.

Two more CpGs (in the MYH6 and NOS1AP genes) achieved genome-wide statistical significance. All 43 differentially methylated sites in the comparison of never smokers versus current smokers were hypomethylated in current smokers and had previously been reported in other populations. However, the overall signature is specific for this Mediterranean population considering that, in this population, we did not observe as statistically significant several of the 25 CpG sites (in HIVEP3 as the hit or in SGIP1, SKI, CUGBP1, SFRS13A, FAM102A, THAP11, SNORD58A, SLC20A1, CYTH4, EDC3, TRAF7, SORBS1 and CTTNl), listed as the 25 most significant genes in the meta-analysis carried out by Joehanes et al. [104] in 16 international cohorts, and detecting 2623 CpGs (annotated to 1405 genes) as statistically significantly differentially methylated in the comparison between current and never smokers. In this meta-analysis, the cg05575921 in the AHRR gene ranked 36th. We observed 24 differentially methylated CpGs in the subgroup analysis comparing former smokers versus current smokers, confirming the reversal of the hypomethylation linked to current smokers [56,104]. In this comparison we reported for the first time a novel association reaching the statistical significance at the EWAS level. It was the cg12093781 site annotated to the KIF26B gene. This CpG site was not included in the updated EWAS Catalog [105]. KIF26B has been identified as an oncogene in several tumors [112] and there is a study examining the relationship between early-life tobacco smoke exposure and genetic variants in this gene on bronchial hyper-responsiveness in asthma [113]. In the subgroup analysis in women, we also discovered a novel CpG associated with tobacco smoking (5 levels). This is the first time that the cg25966498 in the SPATA17 gene has been reported to be involved in such an association. In previous studies, the SPATA17 gene has been linked to germ cell apoptosis in transgenic mice [114], anorexia nervosa [115] and to degenerative diseases [116]. More studies are needed to replicate and to characterize these novel findings in this and other populations. Furthermore, in the sex-specific analysis for tobacco smoking, we explored the association between smoking status and methylation of CpG sites on chromosomes X and Y. In the current EWAS, analysis is typically limited to autosomal chromosomes, and sex chromosomes are frequently overlooked. This is primarily due to the analytical complexities associated with sex chromosome dosage differences between XX and XY individuals, as well as the effect of X-chromosome inactivation on the epigenome [117,118]. However, there is an increasing interest in these chromosomes, and we have obtained some preliminary results in this population. A common finding among the most differentially methylated CpG sites located on chromosome X in the stratified analysis for men and women was for the cg06398113 in the WWC3 gene. The WWC3 gene (cg04224247 site) was previously reported as a novel and top-ranked gene for tobacco smoking in the EPIC study [119]. However more studies are needed to focus on the X and Y chromosomes. Understanding the relationship between the differential methylation at the major CpG sites associated with tobacco smoking and the risk of disease is another important issue. Some studies have examined several associations with cancer, cardiovascular diseases or mortality obtaining mixed results [46,101,120,121,122,123]. A limitation of our study is that we do not have these disease outcomes to analyze the association. Another limitation is the low number of current smokers that does not permit stratifying results according to the number of cigarettes. Likewise, sample size is another limitation to better characterize the modulation by the Mediterranean diet. However, our study has several strengths, including the well-characterized population that we have studied, as well as the homogenous methylation analysis for all the samples, quality control and the use of the 850 K array instead of the 450 K. In addition, we conducted functional enrichment analyses of the differentially methylated CpGs and found strong associations with pathways associated with various cancers, inflammation, insulin metabolism, and cardiovascular diseases, in accordance with previous studies [61,106].

## 5. Conclusions

In this study conducted in a Spanish-Mediterranean population, we characterized the methylation signatures of tobacco smoking at the epigenome-wide level, considering several categories of tobacco smoking and strata. We identified the differentially methylated CpG sites (hypomethylated in current smokers) that are most strongly associated with tobacco smoking in the whole sample and confirmed that, with the exception of one, all have been identified in other populations. As there are dozens of CpGs associated with tobacco use, what distinguishes the methylomic signature of each population is not the identification of new markers, but the significance of each association. In this Mediterranean population, the most strongly associated CpG was the cg21566642 in the 2q37.1 region. In addition, this CpG had the highest discriminative capacity to identify current smokers from never smokers in this population, suggesting its potential use as an exposure biomarker. In subgroup analysis, we discovered some novel differentially methylated CpGs that need to be validated further. Moreover, our exploratory analysis of the modulation of the methylomic effects of tobacco smoking depending on the adherence to the Mediterranean diet revealed that greater adherence to the Mediterranean diet can increase the level of methylation at certain hypomethylated CpG sites in smokers, which is a highly relevant finding and requires additional research and a more in-depth prospective analysis. In light of the fact that previous epidemiological studies leveraging EWAS for tobacco smoking did not analyze dietary modulation, our results encourage further research into such modulations in diverse populations.

## Figures and Tables

**Table 1:** Demographic, clinical and lifestyle characteristics of the study population according to sex.

	Total (n = 414)	Men (n = 186)	Women (n = 228)	*p*
Age (years)	65.1 ± 0.2	63.8 ± 0.4	66.1 ± 0.3	<0.001
BMI (kg/m^2^)	32.3 ± 0.2	32.2 ± 0.3	32.4 ± 0.2	0.440
SBP (mm Hg)	141.9 ± 0.9	143.7 ± 1.4	140.5 ± 1.2	0.076
DBP (mm Hg)	81.0 ± 0.5	82.6 ± 0.7	79.6 ± 0.6	0.002
Total cholesterol (mg/dL)	195.7 ± 1.8	188.1 ± 2.8	202.0 ± 2.3	<0.001
LDL-C (mg/dL)	124.3 ± 1.5	121.5 ± 2.4	126.6 ± 1.9	0.096
HDL-C (mg/dL)	51.6 ± 0.6	47.3 ± 0.8	55.1 ± 0.7	<0.001
Triglycerides ^1^ (mg/dL)	141.3 ± 2.9	139.0 ± 4.0	143.1 ± 4.2	0.488
Fasting glucose (mg/dL)	113.4 ± 1.4	113.7 ± 2.2	113.2 ± 1.8	0.875
Physical activity (MET·min/wk)	1708 ± 78	1941 ± 133	1519 ± 89	0.007
Adherence to MedDiet (17-I) ^2^	8.0 ± 2.8	7.9 ± 2.8	8.1 ± 2.7	0.210
High Adherence MedDiet ^3^ (≥9) (n, %)	172 (41.5)	75 (40.3)	97 (42.5)	0.648
Type 2 diabetes (n, %)	164 (39.6)	75 (40.3)	89 (39.0)	0.790
Never smokers (n, %)	188 (45.4)	32 (17.2)	156 (68.4)	<0.001
Former smokers (>5 years) (n, %)	139 (33.6)	97 (52.2)	42 (18.4)	<0.001
Former smokers (1 to 5 years) (n, %)	25 (6.0)	16 (8.6)	9 (3.9)	<0.001
Former smokers (<1 year) (n, %)	14 (3.4)	11 (5.9)	3 (1.3)	<0.001
Current smokers (n, %)	48 (11.6)	30 (16.1)	18 (7.9)	<0.001
Number of cigarettes smoked per day ^4^	13.2 ± 1.1	14.4 ± 1.6	11.3 ± 1.4	0.186
Number of years smoked ^4^	38.0 ± 1.6	39.1 ± 2.1	36.3 ± 2.6	0.428
Number of pack-years ^4,5^	24.9 ± 2.4	27.9 ± 3.4	19.9 ± 2.4	0.105

Values are mean ± SE for continuous variables and number (%) for categorical variables. BMI: body mass index; SBP: systolic blood pressure; DBP: diastolic blood pressure; LDL-C: low-density lipoprotein cholesterol; HDL-C: high-density lipoprotein cholesterol; MET: Metabolic Equivalent. 1 MET is equivalent to kcal·kg^−1^ · h^−1^, the oxygen cost of sitting quietly measured as 3.5 mL/kg/min; min: minute; wk: week; *p*: *p*-value for the comparisons (means or %) between men and women. Student’s *t* test was used to compare means and Chi squared tests were used to compare categories. ^1^ Triglycerides was ln-transformed for statistical testing. ^2^ Quantitative 17-item (17-I) questionnaire for adherence to Mediterranean diet. ^3^ High Adherence to MedDiet (17-I) ≥ 9 points. ^4^ In current smokers. ^5^ Pack-years = (Number of cigarettes smoked per day/20) × number of years the participant has smoked.

**Table 2:** Top differentially methylated CpGs for smoking status (5 levels) ^1^ ranked by smallest *p*-value after multivariate adjustment ^2^.

CpG Site	Gene Symbol	Chr	Position ^3^	Included in 450 K ^4^	*p*	*r*
cg21566642		2	233284661	Y	2.20 × 10^−32^	−0.548
cg01940273		2	233284934	Y	1.02 × 10^−19^	−0.435
cg14391737	PRSS23	11	86513429	N	8.13 × 10^−19^	−0.425
cg17739917	RARA	17	38477572	N	2.30 × 10^−18^	−0.420
cg21911711	F2RL3	19	16998668	N	6.15 × 10^−17^	−0.403
cg18110140		15	75350380	N	2.12 × 10^−15^	−0.384
cg19572487	RARA	17	38476024	Y	4.26 × 10^−15^	−0.381
cg24859433		6	30720203	Y	6.33 × 10^−15^	−0.378
cg17287155	AHRR	5	393347	Y	2.94 × 10^−14^	−0.370
cg00475490	PRSS23	11	86517110	N	7.08 × 10^−14^	−0.364
cg09935388	GFI1	1	92947588	Y	8.22 × 10^−14^	−0.363
cg05575921	AHRR	5	373378	Y	1.00 × 10^−13^	−0.362
cg15342087		6	30720209	Y	1.78 × 10^−12^	−0.344
cg04551776	AHRR	5	393366	Y	3.50 × 10^−12^	−0.340
cg25189904	GNG12	1	68299493	Y	4.27 × 10^−12^	−0.339
cg03636183	F2RL3	19	17000585	Y	6.36 × 10^−12^	−0.336
cg26703534	AHRR	5	377358	Y	9.58 × 10^−12^	−0.334
cg25648203	AHRR	5	395444	Y	1.08 × 10^−11^	−0.333
cg01901332	ARRB1	11	75031054	Y	3.21 × 10^−11^	−0.325
cg27241845		2	233250370	Y	5.77 × 10^−11^	−0.321
cg19859270	GPR15	3	98251294	Y	8.02 × 10^−11^	−0.319
cg16841366		2	233286192	N	1.04 × 10^−10^	−0.317
cg23161492	ANPEP	15	90357202	Y	1.17 × 10^−10^	−0.316
cg02978227		3	98292027	N	1.04 × 10^−9^	−0.300
cg11660018	PRSS23	11	86510915	Y	1.44 × 10^−9^	−0.298
cg14580211	C5orf62	5	150161299	Y	1.66 × 10^−9^	−0.297
cg11556164	LRRN3	7	110738315	Y	2.00 × 10^−9^	−0.296
cg12806681	AHRR	5	368394	Y	2.96 × 10^−9^	−0.293
cg05086879	MGAT3	22	39861490	N	4.79 × 10^−9^	−0.289
cg05284742	ITPK1	14	93552128	Y	6.54 × 10^−9^	−0.286
cg17738628		15	67155520	N	7.50 × 10^−9^	−0.285
cg21611682	LRP5	11	68138269	Y	8.72 × 10^−9^	−0.284
cg07339236	ATP9A	20	50312490	Y	1.35 × 10^−8^	−0.281
cg24838345	MTSS1	8	125737353	Y	1.54 × 10^−8^	−0.280
cg19554457	NUDT4	12	93774772	Y	1.61 × 10^−8^	−0.279
cg25305703		8	128378218	Y	1.87 × 10^−8^	−0.278
cg04535902	GFI1	1	92947332	Y	2.30 × 10^−8^	−0.276
cg09945032		3	38871019	N	2.59 × 10^−8^	−0.275
cg07986378	ETV6	12	11898284	Y	2.79 × 10^−8^	−0.275
cg08714510	UXS1	2	106755481	N	5.78 × 10^−8^	−0.268
cg15533935	NUDT4P2	12	93774767	N	6.20 × 10^−8^	−0.268
cg16794579	XYLT1	16	17562419	Y	6.28 × 10^−8^	−0.268
cg00310412	SEMA7A	15	74724918	Y	7.04 × 10^−8^	−0.267
cg05221370	LRRN3	7	110738836	Y	7.13 × 10^−8^	−0.267
cg15394081	LMO2	11	33893330	N	8.31 × 10^−8^	−0.265
cg06321596	XYLT1	16	17562960	Y	8.61 × 10^−8^	−0.265

^1^ Five levels of smoking status were considered as an ordinal variable: Never smoker, former smoker, smoker > 5 years, former smoker 1 to 5 years, former smoker < 1 year, and current smoker. ^2^ Models were adjusted for sex, age, diabetes, body mass index, batch effect and leukocyte cell-types (n = 414). ^3^ Position is expressed in values from the Genome Reference Consortium Human Build 37 (GRCh37)-hg19. ^4^ Indicates if the cpg was present (Y) or not (N) in the Illumina 450 K methylation array. Chr: chromosome; *p*: Multivariate adjusted *p*-values; *r*: partial correlation coefficient. The GRCh37/hg19 assembly has been used for the Position values.

**Table 3:** Top-ranked (15) differentially methylated CpGs for smoking status (3 categories) ^1^: (A) and for never smokers (N) relative to current smokers (C) ^2^; (B) ranked by smallest *p*-value after multivariate adjustment ^3^.

A-Smoking (3 Categories)				B-Never (N) Versus Current Smokers (C)				
CpG Site	Gene Symbol	Chr	*p*^4^ (3 Categories)	CpG Site	Gene Symbol	Chr	*p*^5^ (N vs. C)	Beta Difference ^6^
cg21566642		2	1.39 × 10^−29^	cg21566642		2	1.68 × 10^−30^	0.143
cg14391737	PRSS23	11	6.95 × 10^−20^	cg01940273		2	1.30 × 10^−20^	0.097
cg01940273		2	1.44 × 10^−19^	cg14391737	PRSS23	11	1.71 × 10^−17^	0.107
cg17739917	RARA	17	3.44 × 10^−16^	cg17739917	RARA	17	5.13 × 10^−17^	0.112
cg21911711	F2RL3	19	8.14 × 10^−16^	cg21911711	F2RL3	19	8.76 × 10^−17^	0.085
cg00475490	PRSS23	11	9.66 × 10^−16^	cg00475490	PRSS23	11	1.73 × 10^−15^	0.046
cg24859433		6	1.96 × 10^−14^	cg24859433		6	2.29 × 10^−15^	0.048
cg18110140		15	2.36 × 10^−13^	cg18110140		15	3.04 × 10^−14^	0.094
cg09935388	GFI1	1	5.94 × 10^−13^	cg09935388	GFI1	1	6.54 × 10^−14^	0.105
cg25648203	AHRR	5	9.21 × 10^−13^	cg19572487	RARA	17	1.78 × 10^−13^	0.073
cg25189904	GNG12	1	1.46 × 10^−12^	cg25189904	GNG12	1	1.90 × 10^−13^	0.110
cg19572487	RARA	17	1.55 × 10^−12^	cg25648203	AHRR	5	5.67 × 10^−13^	0.065
cg26703534	AHRR	5	2.44 × 10^−11^	cg01901332	ARRB1	11	3.61 × 10^−12^	0.078
cg01901332	ARRB1	11	2.63 × 10^−11^	cg03636183	F2RL3	19	4.54 × 10^−12^	0.132
cg03636183	F2RL3	19	3.84 × 10^−11^	cg17287155	AHRR	5	7.31 × 10^−12^	0.029

^1^ Three categories for tobacco smoking were considered: Never smoker, former smoker, and current smoker (n = 414). ^2^ Never smokers (n = 188) were compared to current smokers (n = 48). ^3^ Models were adjusted for sex, age, diabetes, body mass index, batch effect and leukocyte cell-types. ^4^ *p*-value for the categorical variable (3 categories). Multivariate adjusted *p*-values. ^5^ *p*-value for the comparisons between Never and Current smokers. Multivariate adjusted *p*-values. ^6^ Beta difference for methylation comparing never versus current smokers. Chr: chromosome; N vs. C: comparisons between Never (N) and Current (C) smokers.

**Table 4:** Top-ranked (15) differentially methylated CpGs for former smokers (F) relative to current smokers (C) ranked by smallest *p*-value after multivariate adjustment ^1^.

CpG Site	Gene Symbol	Chr	*p*^2^ (F vs. C)	Beta Difference ^3^
cg21566642		2	2.51 × 10^−16^	0.0935
cg01940273		2	8.57 × 10^−13^	0.0706
cg25648203	AHRR	5	2.39 × 10^−11^	0.0563
cg26703534	AHRR	5	1.20 × 10^−10^	0.0516
cg05086879	MGAT3	22	1.82 × 10^−10^	0.0452
cg18110140		15	7.19 × 10^−10^	0.0719
cg21911711	F2RL3	19	1.49 × 10^−9^	0.0588
cg11556164	LRRN3	7	1.66 × 10^−9^	0.0355
cg25189904	GNG12	1	2.65 × 10^−9^	0.0824
cg23161492	ANPEP	15	3.85 × 10^−9^	0.0631
cg17739917	RARA	17	8.78 × 10^−9^	0.0714
cg04551776	AHRR	5	1.21 × 10^−8^	0.0440
cg24859433		6	1.38 × 10^−8^	0.0332
cg01901332	ARRB1	11	1.67 × 10^−8^	0.0607
cg05284742	ITPK1	14	1.71 × 10^−8^	0.0437

^1^ Models were adjusted for sex, age, diabetes, body mass index, batch effect and leukocyte cell-types. ^2^ *p*-value for the comparisons between former (n = 178) and current smokers (n = 48). Multivariate adjusted *p*-values. ^3^ Beta difference for methylation comparing former versus current smokers. Chr: chromosome; F vs. C: comparisons between former (F) and current (C) smokers.

**Table 5:** Pathway enrichment of methylation of top-ranked sites, obtained in the epigenome-wide methylation analysis of smoking (5 levels), based on the Kyoto Encyclopedia of Genes and Genomes (KEGG). The pathway names are ranked according to the smallest *p*-values and corrected for Bonferroni.

Pathway Name	Enrichment Score	Enrichment *p* ^1^	Bonferroni (Enrichment *p*) ^2^
Parathyroid hormone synthesis, secretion and action	26.912	2.05 × 10^−12^	3.90 × 10^−10^
VEGF signaling pathway	22.448	1.78 × 10^−10^	3.39 × 10^−8^
Morphine addiction	21.911	3.05 × 10^−10^	5.80 × 10^−8^
Endocrine resistance	20.622	1.11 × 10^−9^	2.10 × 10^−7^
Chemokine signaling pathway	18.663	7.85 × 10^−9^	1.49 × 10^−6^
Phospholipase D signaling pathway	18.406	1.01 × 10^−8^	1.93 × 10^−6^
Non-small cell lung cancer	18.024	1.49 × 10^−8^	2.83 × 10^−6^
Glioma	17.219	3.33 × 10^−8^	6.32 × 10^−6^
Cholinergic synapse	17.090	3.78 × 10^−8^	7.19 × 10^−6^
Hepatocellular carcinoma	15.770	1.42 × 10^−7^	2.69 × 10^−5^
Human cytomegalovirus infection	15.279	2.32 × 10^−7^	4.40 × 10^−5^
ErbB signaling pathway	15.148	2.64 × 10^−7^	5.02 × 10^−5^
Relaxin signaling pathway	14.613	4.51 × 10^−7^	8.56 × 10^−5^
Transcriptional misregulation in cancer	13.791	1.03 × 10^−6^	1.95 × 10^−4^
Small cell lung cancer	13.644	1.19 × 10^−6^	2.26 × 10^−4^
Apelin signaling pathway	13.353	1.59 × 10^−6^	3.02 × 10^−4^
Pathways in cancer	13.329	1.63 × 10^−6^	3.09 × 10^−4^
Fc gamma R-mediated phagocytosis	12.986	2.29 × 10^−6^	4.36 × 10^−4^
Circadian entrainment	12.829	2.68 × 10^−6^	5.09 × 10^−4^
Adrenergic signaling in cardiomyocytes	12.365	4.27 × 10^−6^	8.11 × 10^−4^
GnRH secretion	11.470	1.04 × 10^−5^	1.98 × 10^−3^
Growth hormone synthesis, secretion and action	10.053	4.31 × 10^−5^	8.18 × 10^−3^
Melanoma	10.035	4.38 × 10^−5^	8.33 × 10^−3^
Calcium signaling pathway	9.991	4.58 × 10^−5^	8.70 × 10^−3^
Platelet activation	9.558	7.06 × 10^−5^	1.34 × 10^−2^
Axon guidance	9.510	7.41 × 10^−5^	1.41 × 10^−2^
Pancreatic cancer	9.430	8.03 × 10^−5^	1.53 × 10^−2^
Chronic myeloid leukemia	9.430	8.03 × 10^−5^	1.53 × 10^−2^
Dopaminergic synapse	8.928	1.33 × 10^−4^	2.52 × 10^−2^
Kaposi sarcoma-associated herpesvirus infection	8.742	1.60 × 10^−4^	3.04 × 10^−2^
Bladder cancer	8.386	2.28 × 10^−4^	4.33 × 10^−2^
Chemical carcinogenesis—receptor activation	7.854	3.88 × 10^−4^	7.38 × 10^−2^
GABAergic synapse	7.833	3.96 × 10^−4^	7.53 × 10^−2^
Human immunodeficiency virus 1 infection	7.803	4.09 × 10^−4^	7.76 × 10^−2^
Breast cancer	7.713	4.47 × 10^−4^	8.49 × 10^−2^
Gastric cancer	7.580	5.11 × 10^−4^	9.70 × 10^−2^

^1^*p*-value for the pathway enrichment based on KEGG. ^2^*p*-value for the Bonferroni correction.

**Table 6:** Top differentially methylated CpGs for the epigenome-wide methylation analysis of tobacco smoking status (5 levels) ^1^, in men and women, after multivariate adjustment ^2^.

Men (n = 186)					Women (n = 228)				
CpG Site	Gene Symbol	Chr	*p*	*r*	CpG Site	Gene Symbol	Chr	*p*	*r*
cg21566642		2	2.20 × 10^−15^	−0.561	cg21566642		2	4.07 × 10^−16^	−0.522
cg03636183	F2RL3	19	1.92 × 10^−11^	−0.487	cg14391737	PRSS23	11	2.51 × 10^−12^	−0.458
cg01940273		2	3.73 × 10^−10^	−0.458	cg05575921	AHRR	5	1.55 × 10^−11^	−0.443
cg17287155	AHRR	5	8.37 × 10^−9^	−0.425	cg09935388	GFI1	1	5.80 × 10^−11^	−0.431
cg17739917	RARA	17	1.28 × 10^−8^	−0.420	cg17739917	RARA	17	1.32 × 10^−10^	−0.424
cg14391737	PRSS23	11	3.89 × 10^−8^	−0.407	cg00475490	PRSS23	11	2.57 × 10^−10^	−0.418
cg19572487	RARA	17	9.96 × 10^−8^	−0.396	cg01940273		2	8.28 × 10^−10^	−0.407
cg04551776	AHRR	5	1.37 × 10^−7^	−0.392	cg21911711	F2RL3	19	9.46 × 10^−10^	−0.405
cg15342087		6	1.48 × 10^−7^	−0.391	cg24859433		6	1.94 × 10^−9^	−0.398
cg27241845		2	1.56 × 10^−7^	−0.390	cg25966498	SPATA17	1	9.11 × 10^−9^	−0.383
cg21911711	F2RL3	19	2.96 × 10^−7^	−0.382	cg18110140		15	1.08 × 10^−8^	−0.381
cg18110140		15	1.25 × 10^−6^	−0.363	cg11556164	LRRN3	7	1.65 × 10^−8^	−0.376
cg23161492	ANPEP	15	1.28 × 10^−6^	−0.362	cg19572487	RARA	17	2.51 × 10^−8^	−0.372
cg24090911	AHRR	5	1.32 × 10^−6^	−0.362	cg25648203	AHRR	5	6.15 × 10^−8^	−0.362
cg24859433		6	2.12 × 10^−6^	−0.355	cg25189904	GNG12	1	7.29 × 10^−8^	−0.360
cg01901332	ARRB1	11	3.67 × 10^−6^	−0.348	cg21929649	EFTUD2	17	2.90 × 10^−7^	−0.344

^1^ Five levels of smoking status were considered as an ordinal variable: Never smoker, former smoker, smoker > 5 years, former smoker 1 to 5 years, former smoker < 1 year, and current smoker. ^2^ Models were adjusted for age, diabetes, body mass index, batch effect, leukocyte cell-types and stratified by sex (n = 186 in men and n = 228 in women). Chr: chromosome; *p*: Multivariate adjusted *p*-values; *r*: partial correlation coefficient.

**Table 7:** Top differentially methylated CpGs for the epigenome-wide methylation analysis of tobacco smoking status (5 levels) ^1^, depending on the level of adherence to the Mediterranean diet ^2^, after multivariate adjustment ^3^.

Low Adherence to Mediterranean Diet (n = 242)					High Adherence to Mediterranean Diet (n = 172)				
CpG Site	Gene Symbol	Chr	*p*	*r*	CpG Site	Gene Symbol	Chr	*p*	*r*
cg21566642		2	1.34 × 10^−22^	−0.592	cg21566642		2	5.27 × 10^−10^	−0.474
cg03636183	F2RL3	19	1.15 × 10^−16^	−0.516	cg14391737	PRSS23	11	6.21 × 10^−8^	−0.419
cg01940273		2	3.72 × 10^−15^	−0.494	cg02330394	C6orf52	6	3.96 × 10^−7^	−0.395
cg05575921	AHRR	5	4.31 × 10^−15^	−0.493	cg04569608	PLCB3	11	6.31 × 10^−7^	−0.389
cg24859433		6	5.52 × 10^−15^	−0.491	cg00475490	PRSS23	11	8.17 × 10^−7^	−0.385
cg15342087		6	7.71 × 10^−15^	−0.489	cg14175932		14	1.81 × 10^−6^	−0.374
cg17739917	RARA	17	1.41 × 10^−14^	−0.484	cg26742440	RAB6A	11	1.98 × 10^−6^	−0.372
cg09935388	GFI1	1	4.39 × 10^−13^	−0.459	cg22024876	KBTBD3	11	2.37 × 10^−6^	−0.370
cg19859270	GPR15	3	4.79 × 10^−13^	−0.459	cg02286229		11	2.93 × 10^−6^	−0.367
cg21911711	F2RL3	19	7.56 × 10^−13^	−0.455	cg21619351	SCAF1	19	4.83 × 10^−6^	−0.359
cg27241845		2	5.28 × 10^−12^	−0.440	cg13496340	TIGD7	16	5.06 × 10^−6^	−0.358
cg18110140		15	6.71 × 10^−12^	−0.438	cg26815336		6	7.03 × 10^−6^	−0.353
cg14391737	PRSS23	11	9.53 × 10^−12^	−0.435	cg21911711	F2RL3	19	8.06 × 10^−6^	−0.351
cg19572487	RARA	17	1.34 × 10^−11^	−0.432	cg01697541	TMEM97	17	8.57 × 10^−6^	−0.350
cg25648203	AHRR	5	1.43 × 10^−11^	−0.431	cg08376211	MAP7D1	1	9.11 × 10^−6^	0.349
cg26703534	AHRR	5	1.80 × 10^−11^	−0.430	cg22078572	KDSR	18	9.73 × 10^−6^	−0.348
cg18316974	GFI1	1	7.05 × 10^−11^	−0.418	cg22488975	EDEM3	1	1.11 × 10^−5^	−0.346
cg12806681	AHRR	5	9.23 × 10^−11^	−0.415	cg22692169	LINS1	15	1.11 × 10^−5^	−0.346
cg02978227		3	1.41 × 10^−10^	−0.412	cg01207684	ADCY9	16	1.37 × 10^−5^	−0.343
cg18146737	GFI1	1	1.46 × 10^−10^	−0.411	cg17739917	RARA	17	1.64 × 10^−5^	−0.340
cg25189904	GNG12	1	3.59 × 10^−10^	−0.403	cg16552945	ARHGDIG	16	1.78 × 10^−5^	−0.338
cg17287155	AHRR	5	3.74 × 10^−10^	−0.403	cg00502002	ANAPC16	10	2.03 × 10^−5^	0.336
cg23576855	AHRR	5	7.03 × 10^−10^	−0.397	cg18149653		12	2.23 × 10^−5^	−0.335
cg04551776	AHRR	5	8.71 × 10^−10^	−0.395	cg08870961		12	2.37 × 10^−5^	0.334
cg24838345	MTSS1	8	2.35 × 10^−9^	−0.386	cg19355087	NKX6-2	10	2.38 × 10^−5^	−0.333
cg12876356	GFI1	1	5.04 × 10^−9^	−0.378	cg01940273		2	2.42 × 10^−5^	−0.333
cg07986378	ETV6	12	5.25 × 10^−9^	−0.378	cg16915863	LOC400043	12	2.43 × 10^−5^	0.333
cg11660018	PRSS23	11	5.69 × 10^−9^	−0.377	cg21897843	GAB2	11	2.63 × 10^−5^	0.332
cg09945032		3	1.36 × 10^−8^	−0.368	cg18866308	G3BP1	5	2.82 × 10^−5^	−0.331
cg04535902	GFI1	1	1.44 × 10^−8^	−0.367	cg02048416	DOK1	2	2.89 × 10^−5^	−0.330
cg01901332	ARRB1	11	1.71 × 10^−8^	−0.366	cg10352046		12	2.99 × 10^−5^	−0.330
cg24090911	AHRR	5	2.02 × 10^−8^	−0.364	cg16487464		8	3.10 × 10^−5^	0.329
cg16841366		2	2.60 × 10^−8^	−0.361	cg27072224	DNAJC15	13	3.12 × 10^−5^	0.329
cg19554457	NUDT4	12	3.29 × 10^−8^	−0.359	cg21038620	LAT	16	3.31 × 10^−5^	−0.328
cg14372879		1	5.72 × 10^−8^	−0.353	cg02882774	NDUFA6	22	3.57 × 10^−5^	−0.327
cg25305703		8	6.03 × 10^−8^	−0.352	cg09061824	FLOT1	6	3.60 × 10^−5^	−0.326
cg13681954		2	6.48 × 10^−8^	−0.352	cg08445320		14	3.86 × 10^−5^	0.325
cg17738628		15	7.22 × 10^−8^	−0.350	cg27228559	CASC2	10	4.13 × 10^−5^	−0.324

^1^ Five levels of smoking status were considered as an ordinal variable: Never smoker, former smoker, smoker > 5 years, former smoker 1 to 5 years, former smoker < 1 year, and current smoker. ^2^ Low adherence (<9 points in the P-17 Mediterranean diet score); High adherence (≥9 points in the P-17 Mediterranean diet score). ^3^ Models were adjusted for sex, age, diabetes, body mass index, batch effect and leukocyte cell-types (n = 414). Chr: chromosome; *p*: Multivariate adjusted *p*-values; *r*: partial correlation coefficient.

## Data Availability

Neither the participants’ consent forms nor ethics approval included permission for open access. However, we follow a controlled data-sharing collaboration model, and data for collaborations will be available upon request pending application and approval. Investigators who are interested in this study can contact the corresponding author.

## Supplementary Materials

The following supporting information can be downloaded at: https://www.mdpi.com/article/10.3390/ijerph20043635/s1, Table S1: Glossary: Gene symbol and name of the annotated genes for the top-ranked CpG sites; Table S2: Top differentially methylated CpGs for smoking status (5 levels) ranked by smallest *p*-value in the multivariable model unadjusted for leukocytes; Figure S1: Panel A: Ideogram of the top cg21566642 CpG Methylation site for tobacco smoking; Panel B: Regional plot of EWAS results and co-methylation patterns at the cg21566642 CpG site; Table S3: Top differentially methylated CpGs for smoking status (3 categories): (A) and for never smokers (N) relative to current smokers (C); (B) ranked by smallest *p*-value after multivariate adjustment3 (whole list); Table S4: Top differentially methylated CpGs for former smokers (F) relative to current smokers (C) ranked by smallest *p*-value after multivariate adjustment1 (whole list); Table S5: Gene enrichment results for the top-ranked methylation sites in the epigenome-wide methylation analysis for smoking status (5 levels) carried out using the gene ontology (GO) platform; Table S6: Associations between the top-ranked CpG sites obtained in the epigenome-wide methylation analysis for smoking (5 levels) and the number of cigarettes smoked per day (A), or the number of pack-years (B) in smokers (n = 48); Figure S2: Receiver operating characteristic (ROC) curves of the five top-ranked CpG Methylation sites for tobacco smoking (Table 2) for discriminating current smokers and never smokers; Table S7: Association between tobacco smoking (5 levels) and DNA methylation in cg sites located in chromosome X in women; Table S8: Association between tobacco smoking (5 levels) and DNA methylation in cg sites located in chromosomes X and Y in men; Figure S3: Interactions between Mediterranean diet (analyzed as low and high adherence) and smoking status in determining DNA-methylation in selected CpG sites: (A) cg21566642; (B) cg05575921; (C) cg09936388; and (D) cg01901332.
