# Supplementary material for: DNA-Methylation Signatures of Tobacco Smoking in a High Cardiovascular Risk Population: Modulation by the Mediterranean Diet
Source: Int J Environ Res Public Health. 2023 Feb 18;20(4):3635. doi: 10.3390/ijerph20043635 (PMC9964856; doi:10.3390/ijerph20043635)
Supplement: Supplementary file 1 [file ijerph-20-03635-s001.zip › ijerph-2161943-supplementary.pdf]

International Journal of
Environmental Research and Public Health

MDPI

DNA-methylation signatures of tobacco smoking in a high cardiovascular risk population: modulation by the Mediterranean diet

Rebeca Fernández-Carrión, José V. Sorlí, Eva M Asensio, Eva C Pascual, Olga Portolés, Andrea Alvarez-Sala, Francesc Francès, Judith B Ramírez-Sabio, Alejandro Pérez-Fidalgo, Laura V Villamil, Francisco J Tinahones, Ramon Estruch, Jose M Ordovas, Oscar Coltell and Dolores Corella

SUPPLEMENTARY MATERIAL

INDEX

Table S1. Glossary: Gene symbol and name of the annotated genes for the top-ranked CpG sites. 2

Table S2. Top differentially methylated CpGs for smoking status (5 levels)[1] ranked by smallest p-value[2] in the multivariable model unadjusted for leukocytes. 4

Figure S1. Panel A: Ideogram of the top cg21566642 CpG Methylation site for tobacco smoking; **Panel B**: Regional plot of EWAS results and co-methylation patterns at the cg21566642 CpG site...5

Table S3. Top differentially methylated CpGs for smoking status (3 categories)[1]: (A) and for never smokers (N) relative to current smokers (C)[2]; (B) ranked by smallest p-value after multivariate adjustment[3] (whole list). 7

Table S4. Top differentially methylated CpGs for former smokers (F) relative to current smokers (C) ranked by smallest p-value after multivariate adjustment[1] (whole list)..9

Table S5. Gene enrichment results for the top-ranked methylation sites in the epigenome-wide methylation analysis for smoking status (5 levels)[1] carried out using the gene ontology (GO) platform. The GO function, type, enrichment score, enrichment p-value and the Bonferroni corrected p-value are presented ranked by the smallest p-values.......10

Table S6. Associations between the top-ranked CpG sites obtained in the epigenome-wide methylation analysis for smoking (5 levels) and the number of cigarettes smoked per day (A), or the number of pack-years (B) in smokers (n=48)...11

Figure S2. Receiver operating characteristic (ROC) curves of the five top-ranked CpG Methylation sites for tobacco smoking (Table 2) for discriminating current smokers and never smokers. Area under the curve (AUC), 95 % confidence intervals (CI) and the corresponding p-values are presented. 12

Table S7. Association between tobacco smoking (5 levels) and DNA methylation in cg sites located in chromosome X in women[1]. 13

Table S8. Association between tobacco smoking (5 levels) and DNA methylation in cg sites located in chromosomes X and Y in men[1]. 14

Figure S3. Interactions between Mediterranean diet (analyzed as low and high adherence) and smoking status in determining DNA-methylation in selected CpG sites: (A) cg21566642; (B) cg05575921; (C) cg09936388; and (D) cg01901332. P-values for the interaction terms were adjusted for sex, age, body mass index, diabetes, batch effect and leukocytes. 15

Table S1. Glossary: Gene symbol and name of the annotated genes for the top-ranked CpG sites.

Gene symbol	Gene name
ADCY9	adenylate cyclase 9
AHRR	aryl-hydrocarbon receptor repressor
ANAPC16	anaphase promoting complex subunit 16
ANPEP	alanyl aminopeptidase, membrane
ARHGDIG	Rho GDP dissociation inhibitor (GDI) gamma
ARRB1	arrestin, beta 1
ATP9A	ATPase phospholipid transporting 9A (putative)
C5orf62	Small integral membrane protein 3 (SMIM3)
C6orf52	chromosome 6 open reading frame 52
DNAJC15	DnaJ (Hsp40) homolog, subfamily C, member 15
DOK1	docking protein 1
EDEM3	ER degradation-enhancing alpha-mannosidase-like protein 3
EFTUD2	elongation factor Tu GTP binding domain containing 2]
ETV6	ETS variant transcription factor 6
F2RL3	F2R like thrombin or trypsin receptor 3
G3BP1	G3BP stress granule assembly factor 1
GAB2	GRB2 associated binding protein 2
GFI1	growth factor independent 1 transcriptional repressor
GNG12	G protein subunit gamma 12
GPR15	G protein-coupled receptor 15
ITPK1	Inositol 1,3,4-trisphosphate 5/6-kinase family protein
KBTBD3	kelch repeat and BTB domain containing 3
KCNQ1OT1	KCNQ1 opposite strand/antisense transcript 1
KDSR	3-ketodihydrosphingosine reductase
KIF26B	kinesin family member 26B
LAT	linker for activation of T cells
LINS1	lines homolog 1
LMO2	LIM domain only 2
LOC101060019	long intergenic non-protein coding RNA 1799
LOC400043	long intergenic non-protein coding RNA 2381 (LINC02381)
LRP5	LDL receptor related protein 5
LRRN3	leucine rich repeat neuronal 3
MAP7D1	MAP7 domain containing 1
MGAT3	beta-1,4-mannosyl-glycoprotein 4-beta-N-acetylglucosaminyltransferase
MTSS1	MTSS I-BAR domain containing 1
MYH6	myosin heavy chain 6
NKX6-2	NK6 homeobox 2
NRIP1	nuclear receptor interacting protein 1
NUDT4	nudix hydrolase homolog 4
NUDT4P2	nudix hydrolase 4 pseudogene 2
PLCB3	phospholipase C beta 3
PRSS23	serine protease 23
RAB6A	Ras-related small GTP-binding family protein
RARA	retinoic acid receptor alpha
SCAF1	SR-related CTD-associated factor 1

SEMA7A	semaphorin 7A (John Milton Hagen blood group)
SPATA17	spermatogenesis-associated protein 17
SRP14-AS1	SRP14 divergent transcript]
TIGD7	tigger transposable element derived 7
TMEM97	transmembrane protein 97
UXS1	UDP-glucuronic acid decarboxylase 1
XYLT1	xylosyltransferase 1

International Journal of
Environmental Research
and Public Health

Table S2. Top differentially methylated CpGs for smoking status (5 levels)[1] ranked by smallest p-value[2] in the multivariable model unadjusted for leukocytes.

CpG site	Gene Symbol	Chr	CpG_Island	*p*	*r*
cg21566642	intergenic	2	Island	1.68E-34	-0.5613
cg01940273	intergenic	2	Island	4.92E-20	-0.4370
cg14391737	PRSS23	11	S_Shore	3.37E-19	-0.4280
cg17739917	RARA	17	S_Shelf	1.19E-18	-0.4219
cg21911711	F2RL3	19	N_Shore	1.98E-16	-0.3960
cg24859433	intergenic	6		3.31E-15	-0.3807
cg17287155	AHRR	5		3.68E-15	-0.3801
cg18110140	intergenic	15		1.84E-14	-0.3709
cg19572487	RARA	17	S_Shore	2.33E-14	-0.3696
cg00475490	PRSS23	11		3.14E-14	-0.3678
cg05575921	AHRR	5	N_Shore	1.15E-13	-0.3602
cg09935388	GFI1	1	Island	1.28E-13	-0.3595
cg15342087	intergenic	6		8.96E-13	-0.3476
cg04551776	AHRR	5		4.71E-12	-0.3370
cg25189904	GNG12	1	S_Shore	6.01E-12	-0.3354
cg25648203	AHRR	5		8.30E-12	-0.3333
cg03636183	F2RL3	19	N_Shore	1.07E-11	-0.3316
cg16841366	intergenic	2	S_Shore	1.69E-11	-0.3286
cg19859270	GPR15	3		2.97E-11	-0.3248
cg01901332	ARRB1	11		5.59E-11	-0.3204
cg26703534	AHRR	5	S_Shelf	5.61E-11	-0.3204
cg27241845	intergenic	2	N_Shore	5.77E-11	-0.3202
cg02978227	intergenic	3		7.12E-11	-0.3188
cg12806681	AHRR	5	N_Shore	6.72E-10	-0.3027

[1] Five levels of smoking status were considered as an ordinal variable: Never smoker, former smoker, smoker > 5 years, former smoker 1 to 5 years, former smoker < 1 year, and current smoker.
[2] Models were adjusted for sex, age, diabetes, body mass index, and batch effect (n=414).
Chr: chromosome; p: Multivariate adjusted p-values; r: partial correlation coefficient.

Figure S1. Panel A: Ideogram of the top cg21566642 CpG Methylation site for tobacco smoking; **Panel B**: Regional plot of EWAS results and co-methylation patterns at the cg21566642 CpG site.

Panel A

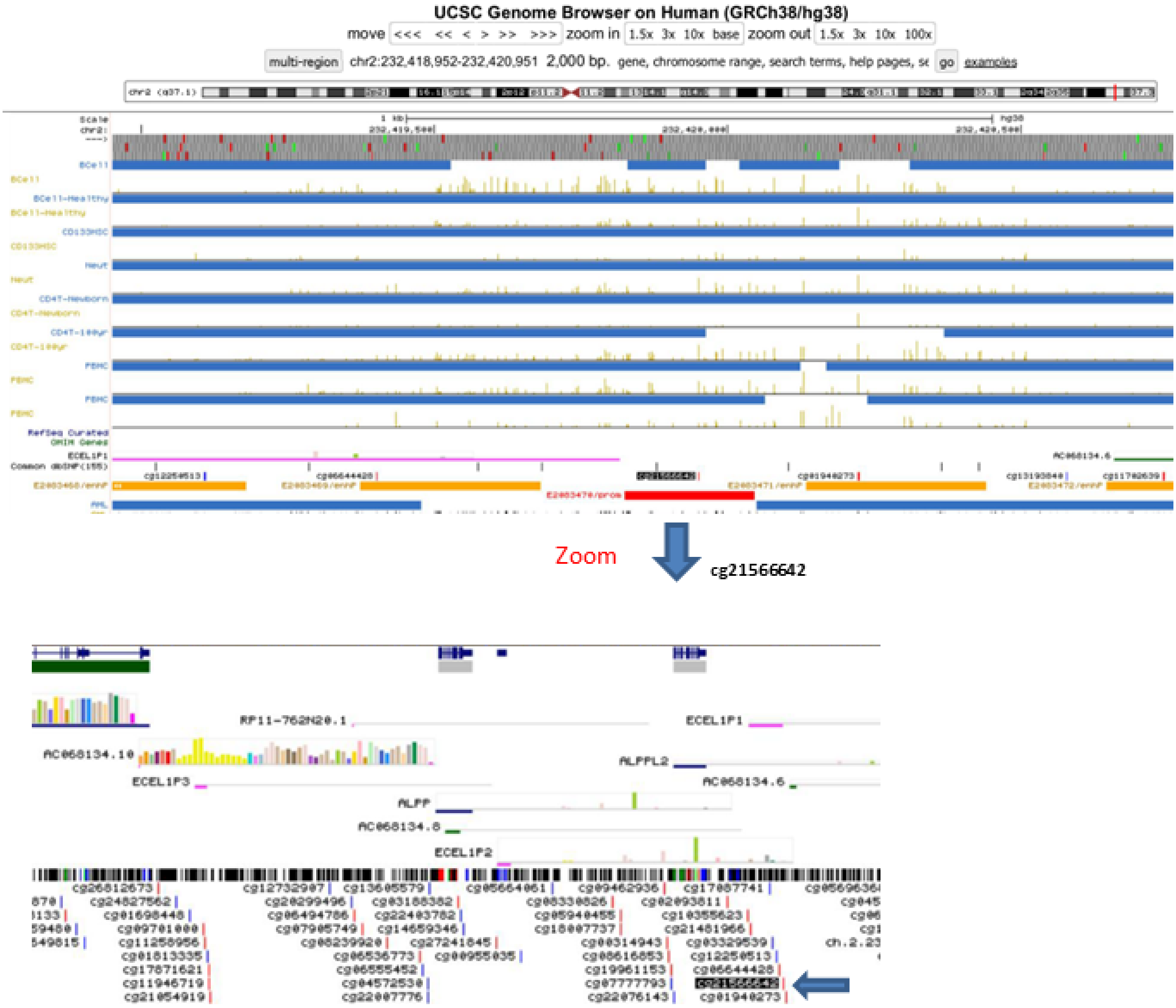

Panel B

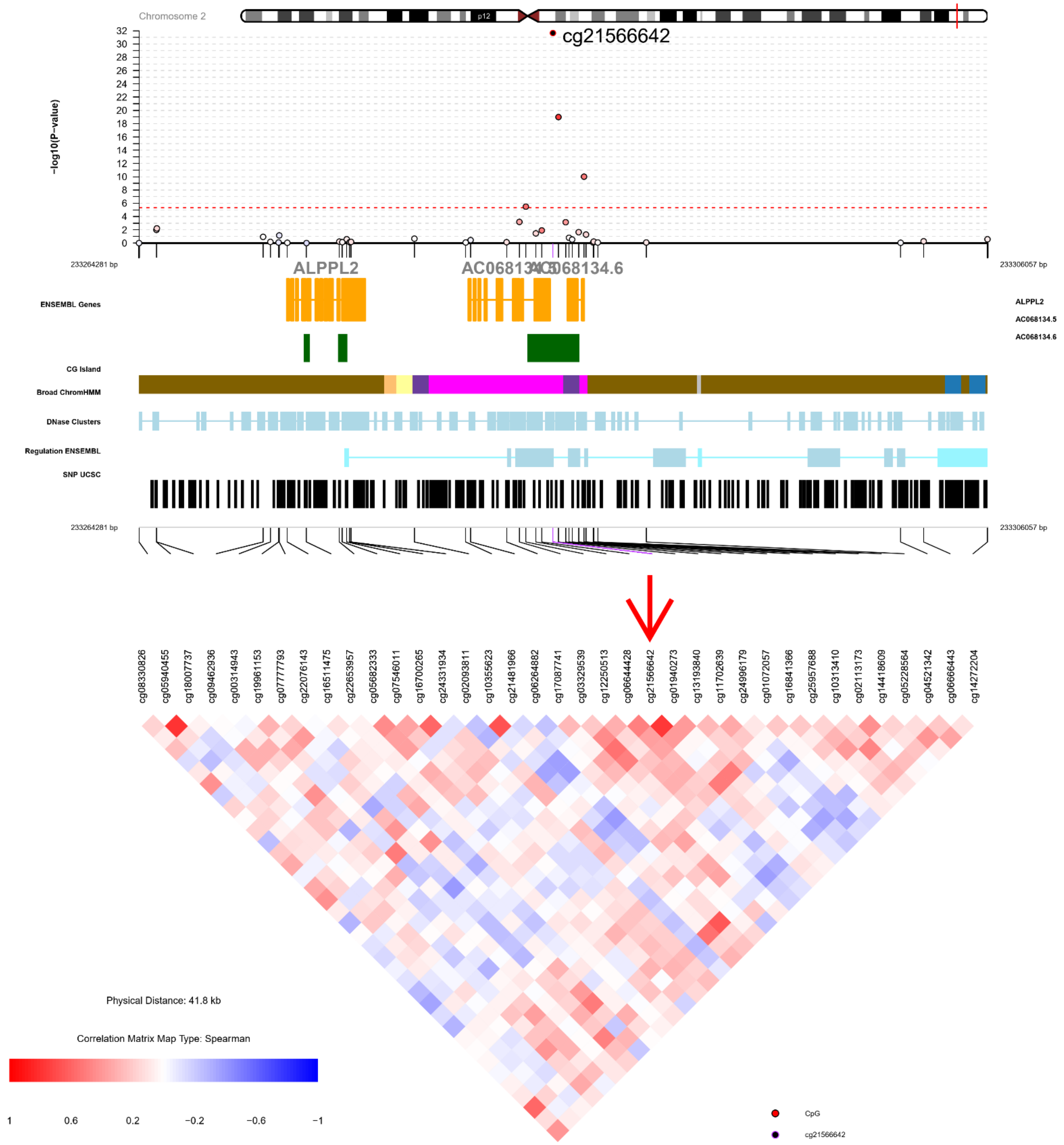

This plot has been depicted using the coMET package: http://comet.epigen.kcl.ac.uk:3838/coMET/.

Table S3. Top differentially methylated CpGs for smoking status (3 categories)[1]: (A) and for never smokers (N) relative to current smokers (C)[2]; (B) ranked by smallest p-value after multivariate adjustment[3] (whole list).

A-Smoking (3 categories)				B-Never (N) versus current smokers (C)				
CpG site	Gene Symbol	Chr	p^4 (3 categories)	CpG site	Gene Symbol	Chr	p^5 (N vs. C)	Beta Difference[6]
cg21566642		2	1.39E-29	cg21566642		2	1.68E-30	0.143
cg14391737	PRSS23	11	6.95E-20	cg01940273		2	1.30E-20	0.097
cg01940273		2	1.44E-19	cg14391737	PRSS23	11	1.71E-17	0.107
cg17739917	RARA	17	3.44E-16	cg17739917	RARA	17	5.13E-17	0.112
cg21911711	F2RL3	19	8.14E-16	cg21911711	F2RL3	19	8.76E-17	0.085
cg00475490	PRSS23	11	9.66E-16	cg00475490	PRSS23	11	1.73E-15	0.046
cg24859433		6	1.96E-14	cg24859433		6	2.29E-15	0.048
cg18110140		15	2.36E-13	cg18110140		15	3.04E-14	0.094
cg09935388	GFI1	1	5.94E-13	cg09935388	GFI1	1	6.54E-14	0.105
cg25648203	AHRR	5	9.21E-13	cg19572487	RARA	17	1.78E-13	0.073
cg25189904	GNG12	1	1.46E-12	cg25189904	GNG12	1	1.90E-13	0.110
cg19572487	RARA	17	1.55E-12	cg25648203	AHRR	5	5.67E-13	0.065
cg26703534	AHRR	5	2.44E-11	cg01901332	ARRB1	11	3.61E-12	0.078
cg01901332	ARRB1	11	2.63E-11	cg03636183	F2RL3	19	4.54E-12	0.132
cg03636183	F2RL3	19	3.84E-11	cg17287155	AHRR	5	7.31E-12	0.029
cg17287155	AHRR	5	5.62E-11	cg04551776	AHRR	5	1.89E-11	0.055
cg04551776	AHRR	5	1.01E-10	cg15342087		6	1.89E-11	0.039
cg15342087		6	1.38E-10	cg26703534	AHRR	5	2.87E-11	0.056
cg05086879	MGAT3	22	1.59E-10	cg05575921	AHRR	5	3.59E-11	0.211
cg05575921	AHRR	5	2.02E-10	cg11660018	PRSS23	11	3.66E-11	0.068
cg23161492	ANPEP	15	2.27E-10	cg23161492	ANPEP	15	8.52E-11	0.072
cg11660018	PRSS23	11	2.96E-10	cg14580211	C5orf62	5	3.47E-10	0.070
cg05284742	ITPK1	14	1.11E-09	cg05284742	ITPK1	14	3.62E-10	0.052
cg11556164	LRRN3	7	1.13E-09	cg05086879	MGAT3	22	5.47E-10	0.048
cg14580211	C5orf62	5	2.72E-09	cg16841366		2	1.11E-09	0.087
cg16841366		2	5.28E-09	cg11556164	LRRN3	7	2.00E-09	0.037
cg19859270	GPR15	3	5.33E-09	cg18146737	GFI1	1	2.07E-09	0.070
cg18146737	GFI1	1	1.41E-08	cg19859270	GPR15	3	2.68E-09	0.023
cg04535902	GFI1	1	1.52E-08	cg04535902	GFI1	1	2.83E-09	0.054
cg17738628		15	3.73E-08	cg16794579	XYLT1	16	1.12E-08	0.053
cg21611682	LRP5	11	3.89E-08	cg27241845		2	1.14E-08	0.076
cg16794579	XYLT1	16	6.77E-08	cg17738628		15	2.27E-08	0.040
cg12806681	AHRR	5	7.01E-08	cg07986378	ETV6	12	2.62E-08	0.068
cg27241845		2	7.85E-08	cg12876356	GFI1	1	2.80E-08	0.077
cg07898952	LMO2	11	8.02E-08	cg00310412	SEMA7A	15	2.96E-08	0.045
cg26673040	LOC101060019	2	8.47E-08	cg02978227		3	3.04E-08	0.020
cg07986378	ETV6	12	1.14E-07	cg06321596	XYLT1	16	4.47E-08	0.065
cg21828716	MYH6	14	1.26E-07	cg21828716	MYH6	14	4.86E-08	0.125
cg00039048	NRIP1	21	1.66E-07	cg07339236	ATP9A	20	5.11E-08	0.035
cg02978227		3	1.94E-07	cg18158149	NOS1AP	1	5.49E-08	0.071
cg12093781	KIF26B	1	1.97E-07	cg12806681	AHRR	5	5.60E-08	0.029
cg12876356	GFI1	1	1.97E-07	cg21611682	LRP5	11	6.06E-08	0.045
cg15394081	LMO2	11	2.05E-07	cg19701538	XYLT1	16	8.65E-08	0.073
cg00310412	SEMA7A	15	2.07E-07	cg08714510	UXS1	2	9.20E-08	0.040

[1] Three categories for tobacco smoking were considered: Never smoker, former smoker, and current smoker (n=414).
[2] Never smokers (n=188) were compared to current smokers (n=48).
[3] Models were adjusted for sex, age, diabetes, body mass index, batch effect and leukocyte cell-types.
[4] p-value for the categorical variable (3 categories). Multivariate adjusted p-values.
[5] p-value for the comparisons between Never and Current smokers. Multivariate adjusted p-values.
[6] Beta difference for methylation comparing never versus current smokers.
Chr: chromosome; N vs. C: comparisons between Never (N) and Current (C) smokers.

Table S4. Top differentially methylated CpGs for former smokers (F) relative to current smokers (C) ranked by smallest p-value after multivariate adjustment[1] (whole list).

CpG site	Gene Symbol	Chr	p^2 (F vs. C)	Beta Difference[3]
cg21566642		2	2.51E-16	0.0935
cg01940273		2	8.57E-13	0.0706
cg25648203	AHRR	5	2.39E-11	0.0563
cg26703534	AHRR	5	1.20E-10	0.0516
cg05086879	MGAT3	22	1.82E-10	0.0452
cg18110140		15	7.19E-10	0.0719
cg21911711	F2RL3	19	1.49E-09	0.0588
cg11556164	LRRN3	7	1.66E-09	0.0355
cg25189904	GNG12	1	2.65E-09	0.0824
cg23161492	ANPEP	15	3.85E-09	0.0631
cg17739917	RARA	17	8.78E-09	0.0714
cg04551776	AHRR	5	1.21E-08	0.0440
cg24859433		6	1.38E-08	0.0332
cg01901332	ARRB1	11	1.67E-08	0.0607
cg05284742	ITPK1	14	1.71E-08	0.0437
cg05575921	AHRR	5	2.35E-08	0.1707
cg09935388	GFI1	1	2.69E-08	0.0762
cg12093781	KIF26B	1	2.81E-08	0.0790
cg21611682	LRP5	11	3.43E-08	0.0433
cg17287155	AHRR	5	4.30E-08	0.0225
cg07898952	LMO2	11	4.68E-08	0.0492
cg26673040	LOC101060019	2	5.87E-08	0.0280
cg19572487	RARA	17	7.13E-08	0.0500
cg00039048	NRIP1	21	8.26E-08	0.0386
cg03636183	F2RL3	19	9.16E-08	0.0948
cg17738628		15	9.27E-08	0.0389
cg25094529	SRP14-AS1	15	9.71E-08	0.0432

[1] Models were adjusted for sex, age, diabetes, body mass index, batch effect and leukocyte cell-types.
[2] p-value for the comparisons between former (n=178) and current smokers (n=48). Multivariate adjusted p-values.
[3] Beta difference for methylation comparing former versus current smokers.
Chr: chromosome; F vs. C: comparisons between former (F) and current (C) smokers.

International Journal of Environmental Research and Public Health

MDPI

Table S5. Gene enrichment results for the top-ranked methylation sites in the epigenome-wide methylation analysis for smoking status (5 levels)[1] carried out using the gene ontology (GO) platform. The GO function, type, enrichment score, enrichment p-value and the Bonferroni corrected p-value are presented ranked by the smallest p-values.

Function	type	Enrichment Score	Enrichment *p*	Bonferroni (Enrichment *p*)
negative regulation of tau-protein kinase activity	biological process	123.12	3.40E-54	1.09E-50
positive regulation of interleukin-13 production	biological process	103.66	9.57E-46	3.08E-42
positive regulation of interleukin-5 production	biological process	82.26	1.89E-36	6.07E-33
perinucleolar compartment	cellular component	74.01	7.19E-33	2.32E-29
regulation of interleukin-13 production	biological process	68.58	1.65E-30	5.32E-27
inositol trisphosphate metabolic process	biological process	61.71	1.58E-27	5.10E-24
regulation of inorganic anion transmembrane transport	biological process	61.71	1.58E-27	5.10E-24
positive regulation of macrophage differentiation	biological process	54.58	1.97E-24	6.34E-21
regulation of endocytic recycling	biological process	48.06	1.34E-21	4.33E-18
negative regulation of granulocyte differentiation	biological process	46.30	7.78E-21	2.50E-17
regulation of protein localization to early endosome	biological process	46.30	7.78E-21	2.50E-17
positive regulation of protein localization to early endosome	biological process	46.30	7.78E-21	2.50E-17
positive regulation of histone H3-K9 acetylation	biological process	46.30	7.78E-21	2.50E-17
regulation of cAMP-dependent protein kinase activity	biological process	42.90	2.33E-19	7.51E-16
cAMP-dependent protein kinase inhibitor activity	molecular function	41.16	1.34E-18	4.30E-15
regulation of anion channel activity	biological process	41.16	1.34E-18	4.30E-15
regulation of interleukin-5 production	biological process	40.71	2.09E-18	6.74E-15
negative regulation of cAMP-dependent protein kinase activity	biological process	37.03	8.25E-17	2.65E-13
regulation of macrophage differentiation	biological process	36.92	9.28E-17	2.99E-13
negative regulation of protein serine/threonine kinase activity	biological process	35.90	2.56E-16	8.25E-13
positive regulation of protein localization to endosome	biological process	33.66	2.42E-15	7.78E-12
cAMP-dependent protein kinase regulator activity	molecular function	33.66	2.42E-15	7.78E-12

[1]: Five levels of smoking status were considered as an ordinal variable: Never smoker, former smoker, smoker > 5 years, former smoker 1 to 5 years, former smoker < 1 year, and current smoker.

Table S6. Associations between the top-ranked CpG sites obtained in the epigenome-wide methylation analysis for smoking (5 levels) and the number of cigarettes smoked per day (A), or the number of pack-years (B) in smokers (n=48).

		**A-Number of cigarettes per day**		**B-Number of pack-years**	
**CpG**	**Gene Symbol**	*r*	*p*	*r*	*p*
cg21566642	intergenic	-0.523	0.0001	-0.516	0.0002
cg01940273	intergenic	-0.418	0.0031	-0.410	0.0038
cg14391737	PRSS23	-0.368	0.0100	-0.531	0.0001
cg17739917	RARA	-0.247	0.0908	-0.344	0.0168
cg21911711	F2RL3	-0.176	0.2308	-0.414	0.0035
cg18110140	intergenic	-0.374	0.0089	-0.450	0.0013
cg19572487	RARA	-0.320	0.0265	-0.332	0.0213
cg24859433	intergenic	-0.328	0.0230	-0.533	0.0001
cg17287155	AHRR	-0.241	0.0992	-0.104	0.4816
cg00475490	PRSS23	-0.269	0.0644	-0.367	0.0102
cg09935388	GFI1	-0.196	0.1821	-0.308	0.0334
cg05575921	AHRR	-0.402	0.0046	-0.509	0.0002
cg15342087	intergenic	-0.382	0.0073	-0.523	0.0001
cg04551776	AHRR	-0.359	0.0122	-0.212	0.1486
cg25189904	GNG12	-0.222	0.1299	-0.352	0.0141
cg03636183	F2RL3	-0.243	0.0967	-0.408	0.0040
cg26703534	AHRR	-0.229	0.1175	-0.359	0.0121
cg25648203	AHRR	-0.365	0.0108	-0.372	0.0092
cg01901332	ARRB1	-0.067	0.6503	-0.333	0.0207
cg27241845	intergenic	-0.238	0.1027	-0.483	0.0005
cg19859270	GPR15	-0.305	0.0351	-0.498	0.0003
cg16841366	intergenic	-0.266	0.0676	-0.401	0.0047
cg23161492	ANPEP	-0.100	0.5010	-0.196	0.1817
cg02978227	intergenic	-0.210	0.1519	-0.343	0.0171
cg11660018	PRSS23	-0.233	0.1108	-0.295	0.0418
cg14580211	C5orf62	-0.196	0.1822	-0.349	0.0150
cg11556164	LRRN3	-0.104	0.4817	-0.187	0.2019
cg12806681	AHRR	-0.051	0.7296	-0.168	0.2534
cg05086879	MGAT3	-0.355	0.0133	-0.316	0.0288
cg05284742	ITPK1	-0.239	0.1013	-0.361	0.0117
cg17738628	intergenic	-0.061	0.6808	-0.262	0.0718
cg21611682	LRP5	-0.173	0.2395	-0.324	0.0247
cg07339236	ATP9A	-0.033	0.8219	0.012	0.9378
cg24838345	MTSS1	-0.082	0.5798	-0.118	0.4260
cg19554457	NUDT4	-0.147	0.3177	-0.210	0.1523

r: Spearman rho; p: p-values.

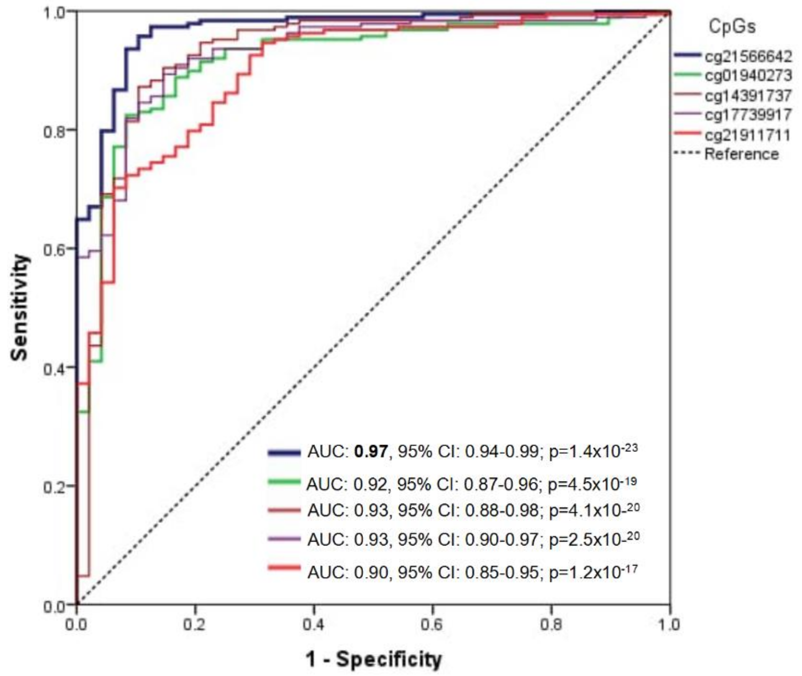

Figure S2. Receiver operating characteristic (ROC) curves of the five top-ranked CpG Methylation sites for tobacco smoking (Table 2) for discriminating current smokers and never smokers. Area under the curve (AUC), 95 % confidence intervals (CI) and the corresponding p-values are presented.

Table S7. Association between tobacco smoking (5 levels) and DNA methylation in cg sites located in chromosome X in women[1].

CpG site	Gene Symbol	Chr	p^2	r^3
cg25433595	XKRX	X	1.96E-05	-0.2893
cg13915481	RAI2	X	2.43E-04	-0.2501
cg08661309	*intergenic*	X	2.76E-04	-0.2479
cg12496847	UPRT	X	3.00E-04	-0.2465
cg07187289	IDH3G	X	3.45E-04	-0.2441
cg24795667	PDHA1	X	3.58E-04	-0.2435
cg07861170	*intergenic*	X	4.38E-04	-0.2399
cg11887001	*intergenic*	X	4.49E-04	-0.2395
cg08626646	*intergenic*	X	4.66E-04	-0.2388
cg18225658	PDZD4	X	5.11E-04	-0.2372
cg13113011	COL4A5	X	6.65E-04	-0.2325
cg00920537	PHF6	X	7.52E-04	-0.2302
cg10860270	ZCCHC5	X	8.11E-04	-0.2289
cg06398113	WWC3	X	8.24E-04	-0.2286
cg25614853	*intergenic*	X	1.04E-03	-0.2243
cg16561205	WWC3	X	1.06E-03	-0.2238
cg11434282	KDM6A	X	1.09E-03	-0.2234
cg08869785	TENM1	X	1.09E-03	-0.2233
cg16488059	LANCL3	X	1.10E-03	-0.2231
cg23192918	MIR505	X	1.10E-03	-0.2231
cg21598868	ACE2	X	1.26E-03	-0.2206

[1.] Models were adjusted for age, diabetes, body mass index, batch effect and leukocyte cell-types.
[2.] p-value for the continuous variable (5 levels) in women. Multivariable adjusted p-values.
[3.] Partial correlation (r) for the continuous variable (5 levels) in women.
Chr: chromosome.

International Journal of
Environmental Research
and Public Health

MDPI

Table S8. Association between tobacco smoking (5 levels) and DNA methylation in cg sites located in chromosomes X and Y in men[1].

CpG site	Gene Symbol	Chr	p^2	r[3]
cg25270201	WWC3	X	9.80E-06	-0.3328
cg25558667	WWC3	X	1.09E-05	-0.3312
cg06398113	WWC3	X	5.27E-05	-0.3058
cg21956484	XPNPEP2	X	5.50E-05	0.3051
cg21207958	SOX3	X	4.17E-04	-0.2685
cg16561205	WWC3	X	4.21E-04	-0.2683
cg18791672	FAM47A	X	5.77E-04	0.2621
cg26739410	FAM47C	X	5.82E-04	0.2619
cg14095687	PNMA3	X	5.94E-04	-0.2615
cg00901375	FAM70A	X	6.11E-04	-0.2610
cg09287749	WWC3	X	6.52E-04	-0.2596
cg09619693	intergenic	X	8.11E-04	-0.2552
cg00379799	intergenic	X	8.52E-04	0.2542
cg16116006	DMD	X	8.73E-04	0.2537
cg02035173	MAGEA4	X	9.03E-04	0.2530
cg07476668	intergenic	X	9.36E-04	0.2523
cg06122016	RBM10	X	1.10E-03	0.2490
cg01831199	RBM41	X	1.20E-03	-0.2471
cg21912308	PRAF2	X	1.42E-03	-0.2435
cg00213853	MPP1	X	1.57E-03	-0.2414
cg23623863	HTR2C	X	1.63E-03	0.2406

[1.]Models were adjusted for age, diabetes, body mass index, batch effect and leukocyte cell-types.
[2.]p-value for the continuous variable (5 levels) in men. Multivariable adjusted p-values.
[3.]Partial correlation (r) for the continuous variable (5 levels) in men.
Chr: chromosome.

International Journal of
Environmental Research and Public Health

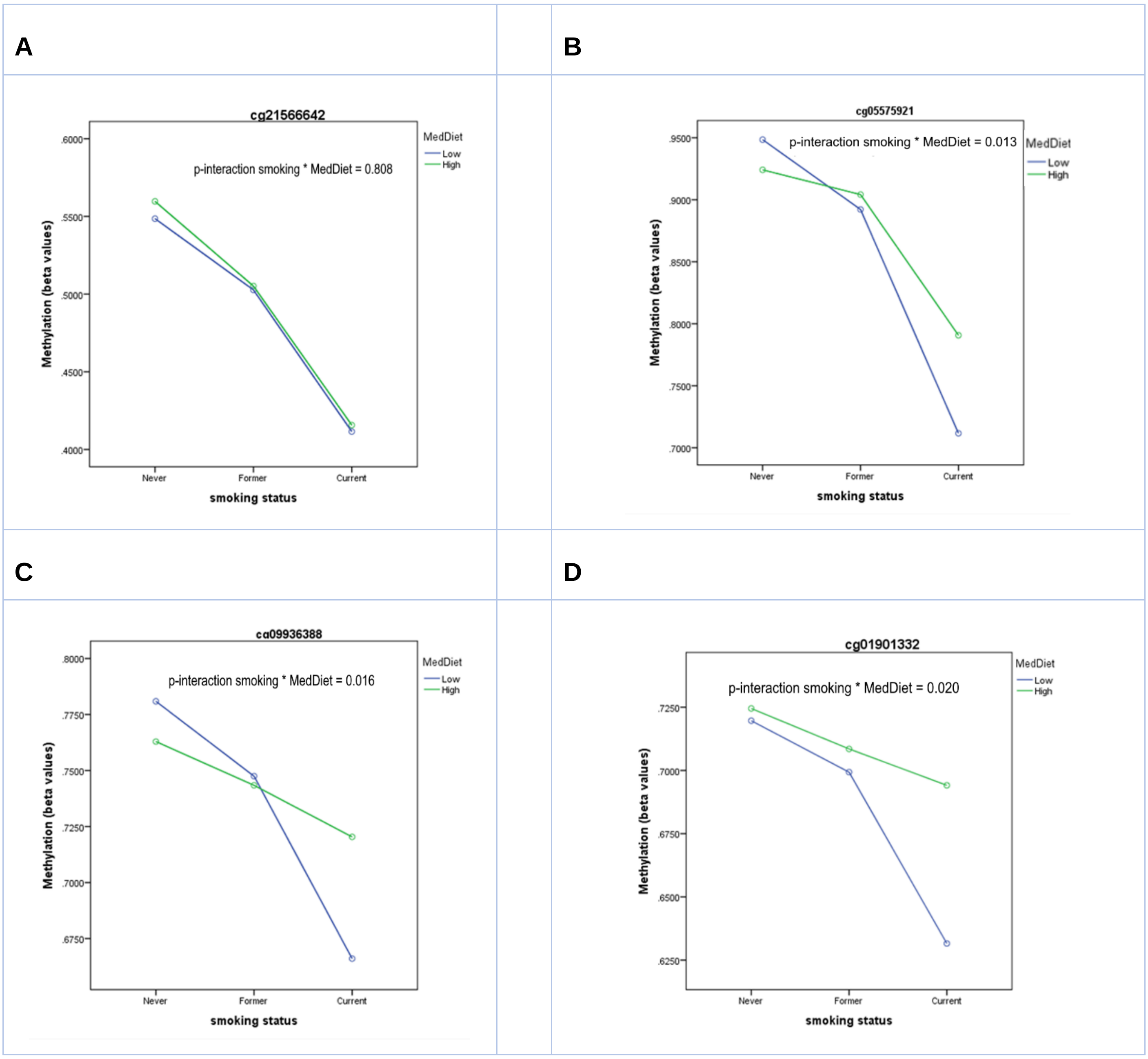

Figure S3. Interactions between Mediterranean diet (analyzed as low and high adherence) and smoking status in determining DNA-methylation in selected CpG sites: (A) cg21566642; (B) cg05575921; (C) cg09936388; and (D) cg01901332. P-values for the interaction terms were adjusted for sex, age, body mass index, diabetes, batch effect and leukocytes.
